# Adaptation of *Saccharomyces* to High Glucose Concentrations and Its Impact on Growth Kinetics of Alcoholic Fermentations

**DOI:** 10.3390/microorganisms12071449

**Published:** 2024-07-17

**Authors:** Marta Ginovart, Rosa Carbó, Xavier Portell

**Affiliations:** 1 Departament de Matemàtiques, Universitat Politècnica de Catalunya-BarcelonaTECH, 08860 Castelldefels, Catalunya, Spain; marta.ginovart@upc.edu; 2 Escola d’Enginyeria Agroalimentària i de Biosistemes de Barcelona, Universitat Politècnica de Catalunya-BarcelonaTECH, 08860 Castelldefels, Catalunya, Spain; rosa.carbo@upc.edu; 3 Departamento de Ciencias Agrarias y del Medio Natural, Escuela Politécnica Superior de Huesca, Universidad de Zaragoza, Ctra. Cuarte s/n, 22071 Huesca, Aragón, Spain

**Keywords:** yeast, aerobic, anaerobic, pre-culture, growth model, kinetic parameters

## Abstract

Prior adaptation of *Saccharomyces cerevisiae* to the fermentation medium ensures its implantation and success in alcoholic fermentations. Fermentation kinetics can be characterized with mathematical models to objectively measure the success of adaptation and growth. The study aims at assessing and comparing two pre-culture procedures using, respectively, one or two adaptation steps, analyzing the impact of different initial glucose concentrations on the fermentation profiles of *S. cerevisiae* cultures, and assessing the performance of three predictive growth models (Buchanan’s, modified Gompertz, and Baranyi and Roberts models) under varied initial glucose concentrations. We concluded that both protocols produced *S. cerevisiae* pre-cultures with similar viability and biomass increase, which suggests that short protocols may be more cost-effective. Furthermore, the study highlights the need of inoculating a high *S. cerevisiae* population to minimize the depletion of dissolved oxygen in the medium and to ensure that glucose is predominantly directed toward the ethanol formation at early fermentative steps. This study shows that the relationship between kinetic parameters is model-dependent, which hinders inter-study comparisons and stresses the need for standardized growth models. We advocate for the generalized use of confidence intervals of the kinetic parameters to facilitate objective inter-study comparisons.

## 1. Introduction

In wine production, spontaneous alcoholic fermentation stands as a microbial process where an array of yeast genera and species are found in the must and participate sequentially thorough the fermentation process. Among these, *Saccharomyces cerevisiae* strains take the lead in the alcoholic fermentation, yet the non-*Saccharomyces* group of yeasts enhances the complexity of the resulting wine [1]. The beneficial roles of both groups are widely acknowledged and can be used to obtain wines with different organoleptic characteristics as a function of fermentation conditions [2]. Nonetheless, the timeframe required for non-*Saccharomyces* yeasts to contribute their distinct microbial influence without causing fermentation delays or halts is not fully characterized. In practice, most winemakers opt to inoculate *Saccharomyces* starters, obtained either from prior spontaneous fermentations within the winery or from commercial sources, aiming at enhancing *Saccharomyces* dominance within the medium, providing better control over the alcoholic fermentation process [3]. Rapid *Saccharomyces* implantation minimizes the risk of wine spoilage, and the use of starter cultures ensures a more consistent product compared to wines originating from spontaneous fermentations [4].

Yeast starters intended for enological applications are predominantly available in the form of dry active yeast. Most commercial strains are pre-conditioned for immediate use, allowing them to be employed right after rehydration. Nonetheless, in order to ensure their success, enologists opt for a prior phase of multiplication and adaptation before the onset of fermentation. Among other aspects, this staged acclimatization approach provides sufficient time for cells to physiologically adapt and acquire the ability to withstand alcohol, sulfites, low pH, and the ability to ferment worts with high initial concentrations of fermentable sugars. This practice of obtaining pre-cultures increases the likelihood of a successful implantation and a fast start of the fermentative process [5].

A contemporary concern among enologists revolves around the phenomenon of grape overripening and the subsequent increase in fermentable sugars in the must, driven by ongoing climate change. This increasingly calls for the adaptation of commercial yeasts to cope with elevated sugar concentrations [6]. A number of works have studied the relationship between the growth and the efficiency of the alcoholic fermentation of *Saccharomyces* in high concentrations of initial carbon source [7]. Nonetheless, rather surprisingly, there is a lack of studies linking the procedure for obtaining the pre-culture and the behavior of the pre-culture in the fermentation medium.

The methodology adopted to conduct the stated studies needs to be considered carefully as well. In that regard, the integration of experimental data with mathematical models facilitates the estimation of growth parameters, fostering the understanding of the fermentation kinetics. A number of modelling approaches, ranging from the Monod or the analogous Michaelis–Menten approaches (see comparison with newer models in [8,9]) and mechanistic individual-based growth models [10] to models dealing with the complex process of phenolic extraction during the red winemaking process [8], are used in the fermentative industry. Models focusing on predicting the growth of the target microorganism are referred to as primary models. Such models can predict and compare *S. cerevisiae* behavior across varying glucose concentrations in fermentation media by estimating key parameters of interest (e.g., initial population, carrying capacity or final population, duration of the adaptation phase, maximum specific growth rate). Insights provided by fitting models to the data help to disentangle the intricate dynamics underlying yeast adaptation, metabolic responses, and fermentation kinetics, providing a comprehensive framework for advancing wine production strategies in response to changing environmental conditions. A number of primary growth models exist, each adapted for different microorganisms and culture media, the approaches of Buchanan et al. [11], Baranyi and Roberts [12], Gibson et al. [13], Zwietering et al. [14], and McKellar [15] being among the most widely used [16]. These models do not consider specifically the inhibition of the substrate but provide estimates of the growth parameters that originate from such inhibition. Once this information has been obtained, the usual approach in predictive modelling is the combination of the information provided by primary growth models under different growth conditions or factors (e.g., concentrations of sugar or nitrate at the beginning of the culture) and building a secondary model, which will allow us to predict growth values (e.g., growth rate or maximum carrying capacity) that can be expected under a given set of experimental conditions or parameters of interest. For instance, D’amato et al. [17] combines the Gompertz model [14] and a secondary model based on a quadratic response surface methodology to characterize the effect of temperature, ammonium, and glucose concentration. Nonetheless, the first key step is choosing a primary growth model suited to the fermentative process at hand.

From this vantage point, the overarching objectives of this study encompass the following: (i) to evaluate and contrast *S. cerevisiae* cultures initiated using inocula derived from two distinct procedures (Pre I and Pre II) differing in the number of adaptation steps; (ii) to analyze the impact of initial glucose concentration (150, 200, and 250 g/L) on the fermentation profiles of the *S. cerevisiae* cultures, and (iii) to assess the performance of three well-known predictive primary growth models (Buchanan’s three-phase linear model, modified Gompertz model, and Baranyi and Roberts model) when applied to cultures grown under varied conditions, and compare the kinetic parameters derived.

## 2. Materials and Methods

### 2.1. Growth Media and Microorganism

A Glucose Peptone Medium (MGP) comprising 3 g/L of yeast extract (Sharlab, Barcelona, Spain), 3 g/L of casein peptone (Sharlab, Barcelona, Spain), and varying quantities of glucose (10, 150, 200, or 250 g/L; Sharlab, Barcelona, Spain) based on the specific test requirements was used. The pH of the medium was carefully adjusted to 3.5 using orthophosphoric acid (Panreac, Barcelona, Spain) and sterilized at 121 °C for 15 min. 

The yeast strain employed was *S. cerevisiae* (LALVIN DV10, LALLEMAND, Edward-Stown, Australia). The yeast was rehydrated in liquid MGP with 10 g/L glucose. Once grown, the purity of the colonies was checked on Sabouraud agar (Sharlab, Barcelona, Spain). At the same time, the cells were stained with methylene blue (Sharlab, Barcelona, Spain) to distinguish dead (stained) from living (unstained) cells and viewed at a magnification of 400X through an Optical microscope (Zeiss, Jena, Germany). To this end, a fraction of the yeast colony was dispersed in 1 mL of Ringer 1/4 saline solution (Sharlab, Barcelona, Spain), 1 mL of methylene blue solution was added, and the resulting solution was homogenized prior to the cell visualization. The strain was maintained on Sabouraud agar at a temperature of 4 °C for approximately one month, and the process of obtaining new yeast was restarted again. 

### 2.2. Pre-Culture Protocols

Two distinct pre-culture protocols for cultivating *S. cerevisiae* inocula, involving either a single-stage (Pre I) or a two-stage approach (Pre II), were examined. Each protocol was tested across three different initial glucose concentrations (150, 200, and 250 g/L), with each combination carried out in triplicate. This resulted in a total of 18 pre-culture incubations (2 × 3 × 3).

In the Pre I protocol, a sole *S. cerevisiae* colony was inoculated into MGP broth that had been directly adjusted to the specific glucose concentration (150, 200, or 250 g/L) under investigation and incubated for 72 h. In contrast, the initial phase of the Pre II protocol mirrored Pre I, but utilized MGP broth containing 10 g/L of glucose. After the initial 72 h period, a volume of 100 µL from the suspension generated in Pre II step 1 was re-inoculated into fresh MGP broth adjusted to either 150, 200, or 250 g/L of glucose and subsequently incubated for an additional 72 h. All stages of the pre-culture process were conducted in 250 mL flasks filled with 100 mL of MGP broth, maintained at a temperature of 27 °C, and stirred using magnetic agitation at a rate of 150 rpm.

### 2.3. Yeast Viability Resulting from Pre-Culture Protocols

Upon completion of the pre-culture phases, a sample of the cell suspension was extracted for the assessment of culture viability. The remaining liquid was preserved at a temperature of 4 °C for a minimum duration of 24 h before initiating the *S. cerevisiae* growth cultures.

Cell suspensions obtained at the end of the pre-culture protocols were analyzed to obtain the number of living, dead, and budding cells using a Neubauer chamber (www.celeromics.com (accessed on 8 February 2024)). Prior to analysis, the cells were stained with methylene blue and viewed at a magnification of 400× through an optical microscope (Zeiss, Jena, Germany).

For the quantification of the viable population, decimal dilutions in Ringer 1/4 saline solution (Sharlab, Barcelona, Spain) were prepared and subsequently placed onto Sabouraud agar. The agar plates were then placed in an incubator for a duration of 48 h at a temperature of 27 °C. The resulting colonies, numbering between 15 and 150, were utilized to quantify the colony forming units (CFU) of *S. cerevisiae*.

### 2.4. Saccharomyces cerevisiae Growth Cultures

#### 2.4.1. Growth Kinetics Assessed via Optical Density

Growth kinetics of the fermentations produced by pre-culture yeast cells were carried out, in triplicate, in microtiter plates with 24 wells (Falcon^®^ 24-well Clear Flat Bottom TC-treated Polystyrene, Corning, NY, USA). Each well was filled with 60 μL of pre-culture and 540 μL of sterile MGP medium adjusted to the glucose concentration tested (150, 200, and 250 g/L). The microtiter plate was incubated at 27 °C and agitated for 45 s before every reading. Growth was monitored every 3 h over a period of 48 h by optical density (OD) measurements (600 nm) using a microtiter plate reader–incubator (Bio-Tek Synergy HT, Agilent, Santa Clara, CA, USA). A calibration of OD data against the plate count method on Sabouraud agar was conducted to transform OD units to colony forming units (CFU/mL) (Figure S1). 

#### 2.4.2. Cell Counting and Biochemical Profiling

A volume of 0.1 mL of the pre-culture suspension was introduced into 1000 mL Pyrex flasks, each containing 600 mL of MGP media with varying glucose concentrations (150, 200, or 250 g/L). These flasks were then incubated at a temperature of 27 °C while being agitated using magnetic stirring over a period of 43.5 h. Each growth condition was conducted in triplicate. 

Sampling was performed at intervals of 1.5 h during the growth cultures. The methodology described previously was followed to determine the count of living cells, dead cells, and budding living cells. Simultaneously, at each sampling time, 3 mL of the culture was frozen for subsequent analysis of glucose and ethanol concentrations.

The dissolved oxygen concentration was monitored using an oxygen electrode (OxyGuard Handy Polaris, Farum, Denmark) at sampling times. The sensitivity of the device was 0.1 mg/L, and calibration was achieved by exposing the probe to air and waiting for temperature stabilization.

For the assessment of ethanol concentration, a gas chromatograph (GC Hewlett–Packard 5890 Series II, Palo Alto, CA, USA) equipped with a flame ionization detector was utilized. An HP-FFAP column (25 m × 0.23 mm) (Agilent, Santa Clara, CA, USA) was employed with nitrogen as the carrier gas. The injection temperature was set at 200 °C, with the oven’s initial temperature at 65 °C and a final temperature of 250 °C, ramping up at a rate of 12 °C per minute. A flow rate of 20 mL/min was maintained for the carrier gas, and a 1 μL sample was injected.

Glucose concentration was determined through high-performance liquid chromatography (HPLC; Beckman, Brea, CA, USA) using a Phenomenex Luna NH2 column (5 μm, 250 mm × 4.6 mm) (www.phenomenex.com (accessed on 8 February 2024)), two Beckman 110B pumps, a Beckman 156 RI detector, and a Hewlett–Packard Series 1100 injector. The mobile phase consisted of acetonitrile/water (75:25, *v/v*) with a flow rate of 1 mL/min. A 20 μL sample was injected. Data analysis was performed using Hewlett–Packard ChemStation software (v. A.06.03), and glucose identification was achieved by comparing the chromatographic pattern to a standard reference.

### 2.5. Modelling of the Growth Kinetics

Microbial growth often shows growth with several phases, which result in a curve that can be fitted by different mathematical functions. Three widely used models were employed for fitting and parameter estimation: (i) the three-phase linear model proposed by Buchanan et al. [11], (ii) the modified Gompertz model introduced by [13] and later reparametrized by [14], and (iii) the Baranyi and Roberts model [12]. The three-phase linear model proposed by Buchanan et al. [11] assumes the growth phases are represented by three lines: one with zero slope for the lag phase, one with slope mumax for the growth phase, and one with zero slope for the stationary phase. The Gompertz and Baranyi and Roberts models can be classified in the sigmoidal curves group. 

The population growth parameters, the duration of the lag phase, the maximum specific growth rate, the initial population density, and the carrying capacity or maximum population density were obtained by fitting these primary growth models.

**Figure 1:**
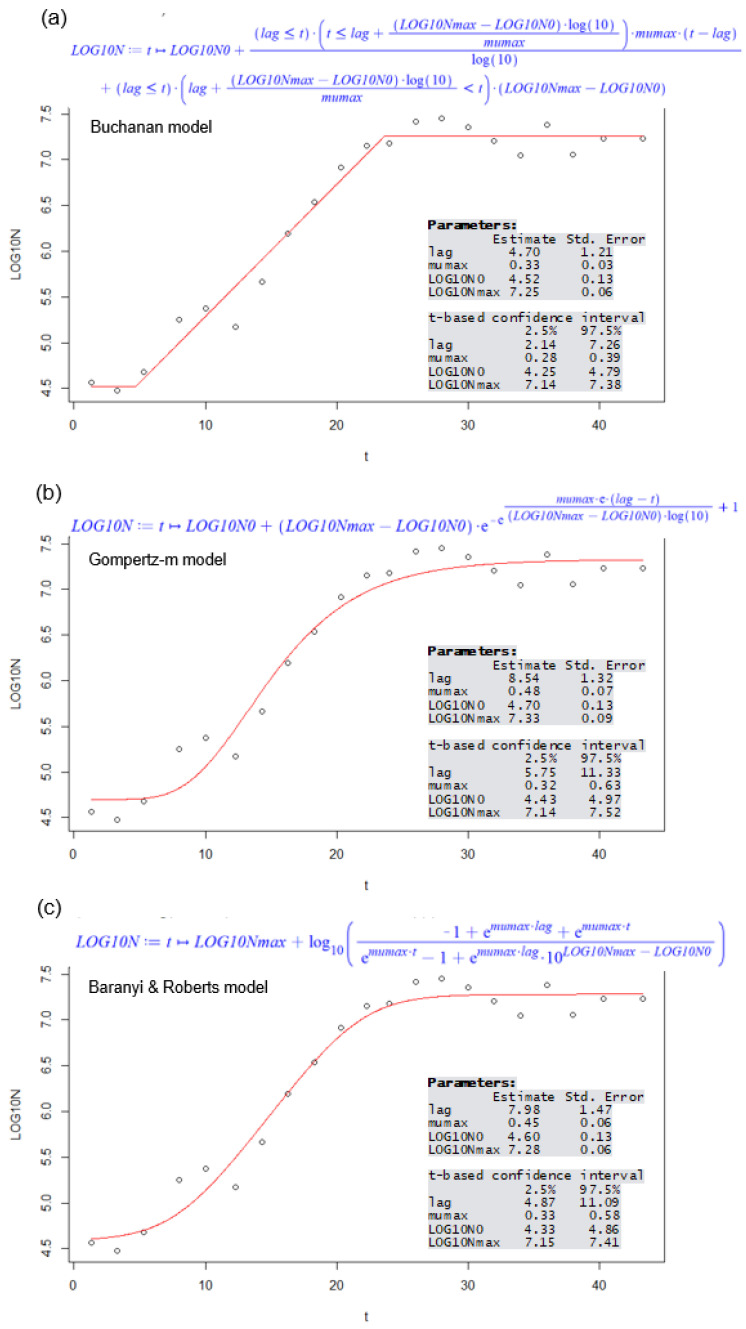
An illustrative example of the R script output fitting the three growth models to experimental data, (**a**) Buchanan, (**b**) Gompertz-m, and (**c**) Baranyi and Roberts, jointly with their corresponding parametrized mathematical expressions. The data subset used corresponds to one of the fermentations with an initial glucose concentration of 250 mg/L. The resulting output showcases the estimated values and their confidence intervals of the four growth kinetics parameters obtained from each model. The identifications of the parameters in these outputs are the duration of the adaptation phase (lag), maximum specific growth rate (mumax), initial population (LOG10N0), and carrying capacity or final population (LOG10Nmax).

Growth data were fitted nonlinearly using nonlinear regression, and the Gauss–Newton algorithm was employed for these fitting procedures. The fitting and parameter estimations were conducted using the free software R (v. 3.6; [18]). The nonlinear regression package nlstools within R [19], along with the nlsMicrobio package dedicated to predictive microbiology [20], were utilized for the growth modeling. These procedures describe the logarithm in base 10 (Log) of the yeast evolution as a function of the time, taking into account the four growth parameters previously mentioned. The formulas of the three primary growth models were provided by nlsMicrobio package [20] and can be found in Figure 1. 

To evaluate the goodness of fit of each model, jointly with visual inspections of the points and the fitted line, the residual standard error (also known as root mean square error) was employed. This metric quantified the average difference between observed data points and the model’s predicted values.

### 2.6. Statistical Analysis

A combination of univariate and bivariate descriptive statistics methods has been employed to provide both numerical and graphical summaries. For deeper insights, inferential statistics methods were utilized. These included:

- Confidence Intervals: To estimate the precision of sample statistics and provide a range of plausible values for population parameters.
- Analysis of Variance (ANOVA): Employed for examining differences among groups, involving both one-factor and two-factor designs. Additionally, interactions between the two factors were assessed.
- Post hoc Tests: Fisher Least Significant Difference method and Tukey’s test were used to compare multiple group means after detecting significant differences through ANOVA.
- Linear Regression Models: Utilized for modelling relationships between variables and making predictions based on these relationships.

These analyses were conducted using Minitab Statistical Software (v. 17.2.1; [21]). The probability level of significance was set at 0.05.

## 3. Results

### 3.1. Evaluation of the Pre-Culture Protocols

#### 3.1.1. Cell Viability under the Pre-Culture Protocols

The viable population (expressed in logarithmic units in base 10, the decimal or common logarithm, Log) and the percentage of living budding cells attained at the conclusion of the pre-cultures prepared using the Pre I and Pre II protocols with three initial glucose concentrations (150, 200, and 250 g/L) can be seen in Table 1 (graphical representations can be found in Figure S2 in the Supplementary Material). 

The Pre I and Pre II protocols, across the three studied initial glucose concentrations, yielded final viable counts ranging from 6 to 7 Log CFU/mL. The lowest and highest mean viable population values were achieved using the Pre I protocol at 150 g/L glucose and 250 g/L glucose, respectively. However, employing an ANOVA with the factors Protocol and Initial Glucose, along with their interaction, revealed no significant differences in mean viable population values concerning the protocol (*p*-value = 0.335) or the initial glucose concentrations (*p*-value = 0.110). Nevertheless, a noteworthy interaction between these two factors was observed (*p*-value = 0.004). Subsequent analysis through Fisher Least Significant Difference method suggested that the mean value obtained from Pre I at 150 g/L glucose significantly differed from mean values obtained using the same protocol at 200 g/L and 250 g/L glucose, as well as from those obtained with Pre II protocol (Table 1).

The recorded average values for the percentage of living budding cells of the pre-cultures ranged from 18% to 36% (Table 1). These observed values fall within a range comparable to those reported in initial *Saccharomyces* kinetic studies conducted by various authors [22,23,24].

Across the three glucose concentrations, procedure Pre II yielded higher average percentages of living cells in budding compared to those achieved with procedure Pre I. Notably, within the same protocol, the average living budding cell percentages were higher when initiated with lower initial glucose concentrations, while they decreased at the 250 g/L initial glucose concentration (Table 1). Nevertheless, upon applying an ANOVA with the factors Protocol and Initial Glucose, along with their interaction, the percentages of living budding cells did not show significant differences among the means of the two protocols (*p*-value = 0.094), or among the three initial glucose concentrations (*p*-value = 0.394), or even among the averages resulting from the interaction of these two factors (*p*-value = 0.847).

#### 3.1.2. Cell Growth Kinetics Produced with the Pre-Culture Protocols

**Figure 2:**
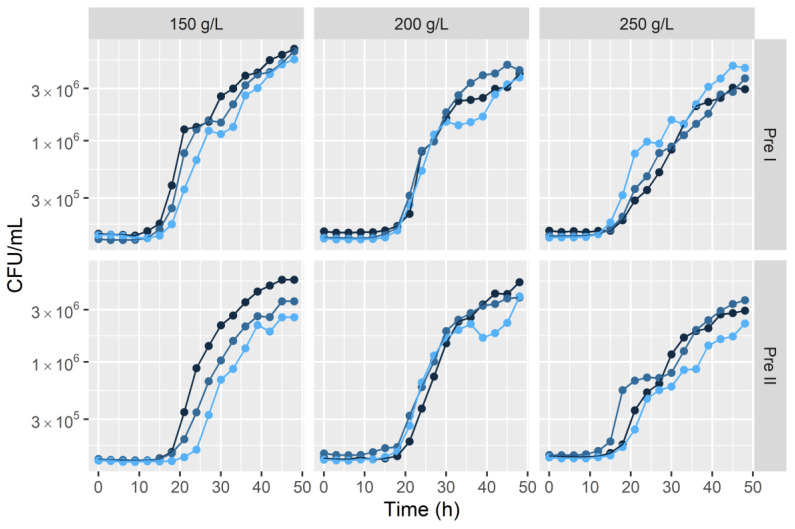
Growth kinetics of *Saccharomyces cerevisiae* in three distinct initial glucose concentrations (150 g/L, 200 g/L, and 250 g/L) inoculated from pre-cultures adhering to Pre I and Pre II protocols. Measurements were obtained by optical density (OD) and subsequently converted into CFU/mL through calibration. The shades of blue denote the repetition.

Figure 2 depicts the growth kinetics of *S. cerevisiae*, utilizing inocula derived from the six pre-cultures. 

Biomass increases at the fermentation step at 48 h, measured as the difference in base 10 logarithmic units between the initial population and the final population (Final Population (Log CFU/mL) − Initial Population (Log CFU/mL)), are outlined in Table 2. These values were derived across the combinations of both protocols and the various glucose concentrations. The highest population count at the 48 h mark was achieved with procedure Pre I at 150 g/L glucose (Mean = 6.80 Log CFU/mL), whereas the lowest count was attained with procedure Pre II at 250 g/L glucose (Mean = 6.46 Log CFU/mL). Notably, these counts corresponded to the highest (1.67) and lowest (1.32) biomass increases, respectively.

The ANOVA performed for the comparison of the means of the biomass increase at 48 h did not show significant differences between protocols (*p*-value = 0.090), nor interaction between the protocol factor and initial glucose (*p*-value = 0.213) (see Table 2). Nonetheless, a significant effect of the initial glucose factor (*p*-value = 0.027) was detected. Tukey’s method for the comparison of the three averages of the biomass increase at 48 h of the initial glucose concentrations (i.e., without considering the effect of nonsignificant factors) identified as significant the difference obtained between that corresponding to the initial glucose of 150 (1.57 Log CFU/mL) and that of 250 (1.37 Log CFU/mL), but did not detect significant differences between 150 and 200, nor between 200 and 250, the average biomass increase at 48 h corresponding to 200 being equal to 1.49 Log CFU/mL.

### 3.2. Biochemical Profiles of Glucose, Dissolved Oxygen, and Ethanol

**Figure 3:**
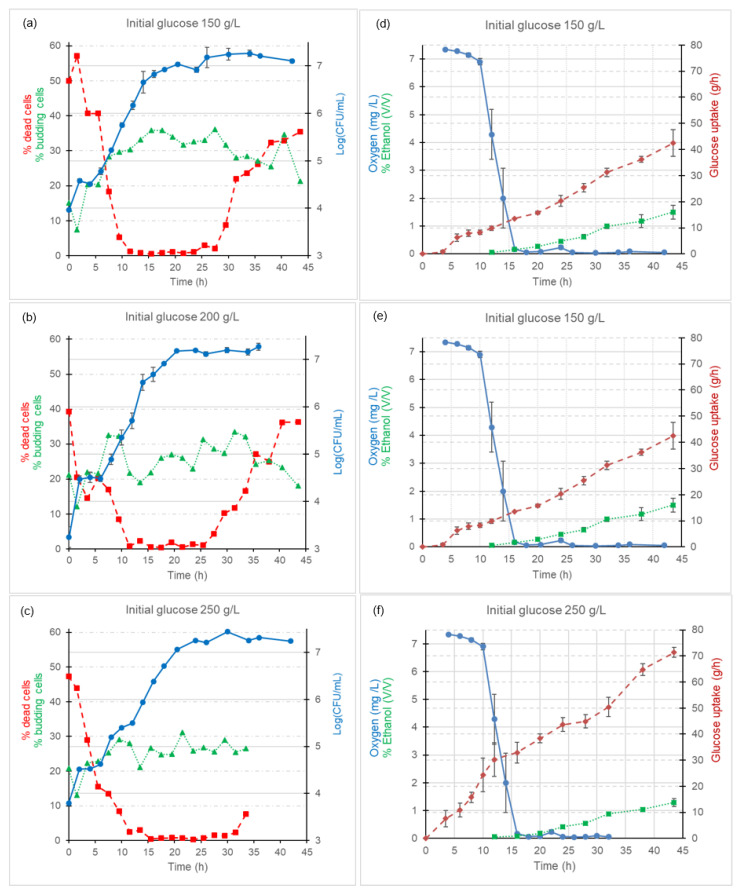
*S. cerevisiae* growth kinetics and biochemical profiles of oxygen, ethanol, and glucose obtained using inoculum from Pre I protocol for the three initial glucose concentrations. In the figure, the mean evolution of the viable population (Log CFU/mL) (circles), percentage of living budding cells (triangles), and dead cells (squares) for initial glucose concentrations (**a**) 150 g/L, (**b**) 200 g/L, and (**c**) 250 g/L are shown. The mean evolution of dissolved oxygen concentration (mg O₂/L) (circles), ethanol concentration (%, *v/v*) (squares), and glucose consumption (g/L) (diamonds) for initial glucose concentrations (**d**) 150 g/L, (**e**) 200 g/L, and (**f**) 250 g/L can also be found. The standard errors of the means are added as error bars.

Figure 3 illustrates the time evolution of the viable population, living budding cell percentage, and dead cell counts during kinetics conducted using pre-cultures prepared through procedure Pre I across three glucose concentrations (150 g/L, 200 g/L, and 250 g/L). The growth kinetics started with initial mean values (±standard deviations) of 3.9 (±0.03) Log CFU/mL for the medium with 150 g/L initial glucose, 3.2 (±0.21) Log CFU/mL for the medium with 200 g/L, and 3.8 (±0.14) Log CFU/mL for the medium with 250 g/L. At the end of the fermentation, the viable population had increased to 7.4 (±0.04), 7.5 (±0.10), and 7.2 (±0.03) Log CFU/mL for media with 150 g/L, 200 g/L, and 250 g/L initial glucose, respectively.

Following the lag phase of the cultures, *Saccharomyces* grew exponentially during the initial 15–20 h of the study. Beyond this period, population growth continued at a reduced rate. Notably, the increase in viable population occurred more rapidly in cultures conducted with lower glucose concentrations compared to those with 250 g/L. Once the peak viable population was attained, it remained stable throughout until the end of the trial (43.5 h). The sustained high viable population levels during the stationary phase align with findings in studies involving fermentations carried out in media with elevated carbon source concentrations [25,26].

The initial proportions of live budding cells were consistently within the range of 15–20% across all kinetics, as depicted in Figure 3a–c. These percentages experienced a nearly 50% reduction and swiftly rebounded and rose to 30–35% in the medium containing 150 g/L glucose, and to 20–30% in media with 200 and 250 g/L glucose concentrations. The living budding cell percentages observed during the lag and early exponential phases were similar to findings from other studies (e.g., [24,27]). Nonetheless, during the advanced exponential phase, these percentages were lower compared to those reported by [27], a discrepancy likely due to the use of culture media with low glucose levels in their work.

Towards the end of the study, and particularly in media containing 150 and 200 g/L glucose, slight declines in the percentages of living budding cells became evident, coinciding with the advanced stationary phase. For the medium with 250 g/L glucose, final experimental data were not available, and the corresponding behavior could not be observed.

The initial proportions of dead cells were consistently between 40% and 50% across all kinetics, as depicted in Figure 3a–c. These proportions exhibited a decline coinciding with the rise in viable cells and living budding cells. However, in kinetics involving 150 g/L and 200 g/L glucose concentrations, the percentages of dead cells experienced a subsequent increase starting around 25 h, coinciding with the stationary phase. Albeit with a certain delay, a similar trend in the increase in dead cell percentage was observed from 30 h in the medium containing 250 g/L glucose.

**Figure 4:**
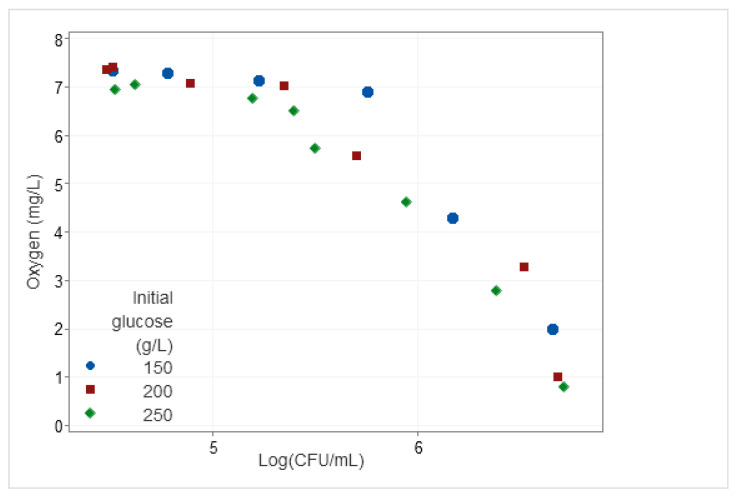
Relationship between dissolved oxygen concentration (mg O₂/L) and viable population (Log CFU/mL) corresponding to the three initial glucose concentrations.

Figure 3d–f shows the progression of dissolved oxygen concentration (mg O₂/L), ethanol concentration (% *v/v*), and glucose consumption (g/L) for cultures conducted across three glucose concentrations (150 g/L, 200 g/L, and 250 g/L). The kinetics started with initial dissolved oxygen concentrations of 7–8 mg/L. These concentrations remained elevated for the initial ten hours, during which oxygen depletion occurred slowly. Subsequently, a significant decline in dissolved oxygen concentrations occurred, reaching values nearly approaching zero between 15 and 18 h from the culture start. This decrease in dissolved oxygen content in the medium (Figure 3d–f) coincided with the advanced exponential growth phase depicted in Figure 3a–c. A discernible relationship between the magnitude of the viable population and the concentration of dissolved oxygen within the environment was evident. As shown in Figure 4, the higher the viable population, the lower the dissolved oxygen concentration found in the media irrespective of the initial glucose concentration tested. The decrease in dissolved oxygen per biomass unit becomes more evident for higher yeast counts.

Glucose consumption took place throughout the entire 43.5 h duration of the study (Figure 3d–f). At the end of the study, glucose utilization reached 28% in the media with initial glucose concentrations of 150 g/L and 250 g/L, and 34% in the medium containing 200 g/L glucose. Notably, in the medium with 200 g/L glucose, a significant reduction in glucose content was observed right from the beginning of the fermentation. For instance, within the initial 6 h, as much as 22 g of glucose was consumed in this medium, while in the same timeframe, 6 g and 11 g were consumed in media with 150 g/L and 250 g/L glucose, respectively. This considerable difference in glucose consumption in the medium with 200 g/L glucose coincided with a more pronounced initial increase in the viable population.

Starting from approximately 10–12 h into the study, the glucose consumption trends in the media with 200 g/L glucose displayed steeper slopes compared to those observed in the media with 150 g/L and 250 g/L glucose concentrations.

Ethanol concentration measurement commenced at the 12 h mark, showing a comparable ethanol production across the three glucose concentrations, approximately amounting to 0.1% (*v/v*) ethanol (Figure 3d–f). From the 12 h point, ethanol concentration exhibited gradual increments, roughly growing by around one-tenth with each successive measurement. By the end of the study (43.5 h), the medium containing 150 g/L glucose had reached an ethanol concentration of 1.5% (*v/v*), the medium with 200 g/L glucose exhibited 1.1% (*v/v*) ethanol content, and the medium with 250 g/L glucose displayed 1.3% (*v/v*) ethanol content.

### 3.3. Influence of the Initial Glucose Concentration on Model Parameterization

Mathematically, the growth models establish the relation between the yeast population as a dependent variable and time as an independent variable. They allow us to derive four kinetic parameters, namely the duration of the adaptation phase, maximum specific growth rate, initial population, and carrying capacity or maximum population. Figure 1 illustrates an exemplar subset of experimental data, showcasing the parameter estimations from the three models along with associated information about estimation precision, point estimates, and corresponding confidence intervals. The full collection of fittings with the three models for all experimental data subsets is available in the Supplementary Material (Table S1 and Figure S3). Across the 27 fittings conducted, the residual standard errors (also known as root mean square errors) ranged from 0.11 to 0.23. This metric is a measure of the extent of deviation between observed and predicted values, providing an average measure of this deviation.

Overall, all models showed a high goodness-of-fit to the data. Visual inspections of the fittings also suggested a good performance with no systematic over- or underestimation of any curve segment. A comparative analysis of residual standard errors through ANOVA yielded a *p*-value of 0.829, indicating no significant differences in goodness-of-fit among the models. Furthermore, no substantial distinctions were identified between initial glucose concentrations (*p*-value = 0.485), and no interaction was noted between model and initial glucose (*p*-value = 0.419).

The mean residual standard errors for the Buchanan, Gompertz-m, and Baranyi and Roberts models were 0.158, 0.164, and 0.155, respectively (graphical representations of the residual standard errors can be accessed in the Supplementary Material, Figure S4). Overall, all three models displayed a similar fit quality across all data subsets. Through the application of these three models, the differentiation of adaptation, exponential, and stationary phases was achieved, facilitating accurate estimation of the corresponding kinetic parameters in the function of the initial glucose concentration in the medium. 

#### 3.3.1. Growth Parameters Obtained with the Microbial Models

Table 3 presents the means, coupled with their associated confidence intervals, for the kinetic parameters linked to initial population, lag phase duration, specific growth rate, and final microbial population estimated by fitting the models of Baranyi and Roberts, Buchanan, and Gompertz-m to the three sets of replicated experimental growth data of the three initial glucose contents tested. 

ANOVA was the statistical method used to compare the average parameter estimates for the four kinetics parameters across the distinct experimental conditions (Table 4).

A two-factor ANOVA with interaction was used to compare estimates of kinetic parameters obtained across various growth profiles with the three models (graphical representation of these results can be found in Figure S5 in the Supplementary Material). 

The ANOVA for the initial population did not reveal any significant difference, neither among initial glucose concentrations nor among the models employed for estimation (Table 4). Additionally, no significant interaction was detected between the factors model and initial glucose (Table 4). The overall mean for the initial population estimated was 4.5 Log CFU/mL. 

Mean lag phase lengths spanned from 4.7 h to 7.1 h. ANOVA analysis found significant differences among lag phase lengths estimated from distinct models and among growth media containing differing initial glucose concentrations (Table 4). Nonetheless, the interaction between the factors mathematical model and glucose concentration was found to be not significant (Table 4). Tukey post hoc test showed significant differences in average lag phase durations between the Gompertz-m and Buchanan kinetic models. Specifically, the Gompertz-m model yielded an average lag of 6.3 h, contrasting with the 5.0 h derived from the Buchanan model. Conversely, employing the Baranyi and Roberts model for lag estimation (6.0 h) did not yield any significant differences compared to either the Buchanan or Gompertz-m models. Furthermore, significant differences between lag times from distinct initial glucose concentrations were identified. Tukey’s test confirmed that the medium containing 150 g/L glucose exhibited a markedly distinct behavior compared to the other two initial concentrations. Specifically, the lag phase duration in the 150 g/L glucose medium amounted to 5 h, while it extended to 6.2 h in the media with an initial glucose concentration of 200 g/L and 250 g/L. 

Mean specific growth rates ranged from 0.37 h^−1^ to 0.69 h^−1^ (Table 3). ANOVA suggested significant differences between specific growth rate averages estimated from distinct mathematical models, from yeast growing in alternative initial glucose concentrations (Table 4). The interaction between the model and glucose factors was, however, not significant (Table 4). Specifically, the Buchanan model yielded a slightly lower average specific growth rate value (0.46 h^−1^) compared to averages derived from the Baranyi and Roberts model (0.58 h^−1^) or the Gompertz-m model (0.60 h^−1^). Nonetheless, the post hoc Tukey’s test could not distinguish among these mean estimates. Further examining the models via the post hoc Fisher test, significant differences were found between the Gompertz-m and Buchanan models. However, the Baranyi and Roberts model did not exhibit significant differences when contrasted with the other two models. Focusing on specific growth rate predicted for media with alternative initial glucose concentrations, Tukey’s test suggested significant differences between the mean specific growth rate values from the medium with an initial glucose concentration of 200 g/L (0.63 h^−1^) and the concentration of 250 g/L (0.45 h^−1^). Conversely, the mean value with 150 g/L glucose could not be distinguished from either of the other two concentrations (0.56 h^−1^).

Mean estimates for the yeast population at the end of the culture (LOG10Nmax) ranged from 7.1 to 7.3 Log CFU/mL ANOVA found no significant differences between estimates obtained from distinct mathematical models, or for the interaction factor (Table 4). However, differences between estimates from distinct initial glucose concentrations were detected (Table 4). Tukey’s test highlighted a marked difference between the mean final population values in media containing 150 g/L and 250 g/L glucose. Specifically, in the medium with 250 g/L glucose, the final population was significantly higher (7.28 Log CFU/mL) compared to that of the medium with 150 g/L glucose (7.18 Log CFU/mL). Nevertheless, neither of them demonstrated a significant difference from the outcome with 200 g/L glucose (7.24 Log CFU/mL).

#### 3.3.2. Correlation between Parameter Estimates

Correlations between kinetic parameters produced by alternative models can be investigated using linear correlation. This analysis aims to capture discernible relationships or trends in the behavior of each model during parameter estimation (Table 5). In addition to the substantial differences in the value of the growth parameter among different models for the same dataset, the analysis suggests that the linear relationship between certain pairs of parameter estimates is not universally consistent, and studied models yield varying degrees of linear relationships between estimated parameters. For instance, while the direct linear relationship between the specific growth rate and the lag phase duration is evident when the Buchanan model is employed, this relationship ceases to hold when parameters are estimated using the Baranyi and Roberts model or the Gompertz-m model. Such divergent behavior across models is also evident when the inverse linear relationship between LOG10N0 and lag is investigated.

The linear regression study conducted between pairs of estimates of the parameters from alternative models can be found in the Supplementary Material. Table S2 provides the coefficients of the linear correlations with their significance, the fitted regression lines, in addition to information about their intercepts and slopes. These outputs allow us to fundamentally conclude whether the estimates for the coefficients of the linear relationship, intercept, and slope can be assumed to be equal to 0 and equal to 1, respectively. From this complementary analysis carried out, it becomes evident that the relationship between an estimate made for a given parameter stands for a different linear equation in each case, which suggest that, for the same dataset, estimates made by a given model cannot be derived directly from estimations conducted with another growth model.

## 4. Discussion

### 4.1. Assessment of the Pre-Culture Protocols

The observed lower viable population count of pre-cultures prepared with procedure Pre I and 150 g/L glucose (Figure 2), in comparison to Pre I pre-cultures with higher glucose concentrations, suggests that pre-cultures with lower glucose concentrations might have transitioned into the stationary phase more rapidly, due to faster growth than in medium with higher glucose concentration [6,7]. The accelerated growth rate may have therefore led to an aged population at sampling, subsequently contributing to lower viable population counts.

The slightly lower, non-statistically significant, counts of the viable population obtained through protocol Pre II can be explained by considering the initial stage of this protocol. As suggested by a preceding study [28], the population likely attained a stationary phase during the first step of the protocol. Consequently, during the subsequent stage of the Pre II protocol, the inoculum reactivation in a high-glucose-concentration environment would have required of an extended lag phase [29]. The reactivation of the Pre II population is likely driven by the proportion of viable budding cells. The only significant differences in biomass increase at 48h are due to glucose and not due to the protocol.

The simultaneous assessment of the viable population and the percentage of living budding cells of the pre-cultures obtained, as well as the biomass increase at 48 h shown by these pre-cultures, enable us to conclude that there are no significant differences between the two protocols tested. In procedure Pre I, a single-stage approach is used, involving simultaneous cell multiplication and adaptation to high glucose concentrations (promoting fermentative growth). Procedure Pre II begins with a phase marked by growth in lower glucose concentrations, fostering biomass production through oxidative metabolism, followed by a second stage favoring fermentative growth under high-glucose conditions [30]. Both protocols yield comparable viable populations, also aligning with findings from further studies such as the one of [7], albeit slightly lower in comparison to multi-stage protocols used in sparkling wine production. These protocols encompass additional complexities, including adaptation to ethanol, atmospheric pressure, and sulfur dioxide, often resulting in longer durations [31].

The current study’s outcomes underline that the studied *S. cerevisiae* strain demonstrates the capacity to grow with similar biomass increase at 48 h using both protocols. Given these observations and considering that protocol Pre I was more resource-efficient and time-effective, fulfillment of the second and third objectives of this study was pursued exclusively using pre-cultures prepared using the Pre I procedure.

### 4.2. Biochemical Profiles of Glucose, Dissolved Oxygen, and Ethanol in Three Initial Glucose Concentrations

Analysis of the shifts in viable population and the percentages of living and budding cells allows the assessment of the extent of adaptation to varying glucose concentrations exhibited by Pre I pre-cultures. The elevated initial dead cell percentage can be explained by the challenging conditions experienced by the pre-cultures before the inoculation, characterized by low dissolved oxygen concentrations (≤1.4 mg/L) and prolonged exposure (24 h) at temperatures not exceeding 4 °C. 

The rise in live budding cells became apparent during the lag phase and within the initial 5–8 h of the logarithmic growth phase. The decline in the percentage of living budding cells observed in the second control aligns with the findings of [24], who attributed it to gem cells from the inoculum that had attained a critical size and detached from the mother cell. The elevated percentages of living budding cells were consistently sustained, with occasional fluctuations, throughout the remainder of the exponential phase and a substantial portion of the stationary phase. The lower percentages of budding cells in media containing 200 g/L and 250 g/L glucose, in contrast to the medium with 150 g/L glucose, can be attributed to the more rapid glucose consumption rate in the former, which adversely impacts the budding cell percentage [23]. Additionally, in the medium containing 250 g/L glucose, growth is hampered due to the increased osmotic pressure.

A previous study by our team [32], utilizing the same medium with an initial glucose concentration of 10 g/L, reported maximal living budding cell percentages during the exponential phase, which subsequently decreased during the stationary phase. This highlights the influence of glucose concentration in the medium on the dynamics of living budding cell fractions during different growth stages. Consequently, suggesting that relying solely on this parameter, even though reportedly well correlated with cellular growth activity [33], does not allow differentiating *Saccharomyces* growth stages, as opposed to morphometric variables such as cell size, which have demonstrated such discriminative potential [32].

Within the initial 12–15 h of the experiment, the culture transitioned from aerobic to anaerobic conditions due to the metabolic activity of *Saccharomyces* itself. This metabolic shift led to an augmentation of the viable population while causing a reduction in dissolved oxygen levels within the medium [34]. Notably, the data obtained underscore a robust correlation between viable cell counts and dissolved oxygen concentrations (Figure 4).

*Saccharomyces* is a Crabtree yeast that, under aerobic conditions and in the presence of elevated glucose concentrations, suppresses its respiratory pathway, and carbon predominantly follows the fermentative route. Nevertheless, even in the presence of oxygen, there exists a discernible carbon flux between respiration and fermentation [35]. In our experiment, the initial production of ethanol was likely minimal due to the relatively low viable population. Moreover, a portion of the ethanol that was being generated might have evaporated [36]. Initial ethanol quantification commenced when approximately 15.7 g/L, 36.7 g/L, and 38.4 g/L of glucose had been consumed in media with initial glucose concentrations of 150 g/L, 200 g/L, and 250 g/L, respectively. This point coincided with a nearly complete depletion of dissolved oxygen and viable population counts reaching or exceeding 5 Log CFU/mL. It is worth noting that roughly 17 g/L of glucose is typically required for the formation of 1% (*v/v*) ethanol [37]. As such, most of the carbon metabolized up to that point was likely directed towards generating new biomass [7,38].

In the final phase of the study, spanning the last 8–10 h of the cultures, even when substantial glucose concentrations, anaerobic conditions, and high viable population counts persisted, a marked increase in ethanol production occurred, which has also been reported by other authors [39]. This may suggest that the Pasteur effect (i.e., ethanol production under anaerobic conditions and high carbon source) has dominated glucose metabolism until the end of the study. This pattern aligns well with the fermentative process typically observed in wines, as described by [40]. Starting at the 43.5 h mark and with the population entering the stationary phase, alcoholic fermentation would continue until peak ethanol levels are reached.

### 4.3. Impact of Model Selection on the Estimation of Saccharomyces cerevisiae Growth Parameters

Three primary deterministic models employing a common set of parameters were evaluated in this study, namely the Buchanan, Gompertz-m, and the Baranyi and Roberts models. Analyzing the goodness-of-fit results achieved, showing similar performance for all the growth functions, none of the models could be selected as being superior to the others. In a similar comparison of nine growth models (including the ones tested here) for fitting the growth of the algae *Dunaliella tertiolecta*, the Baranyi and Roberts and Gompertz-m models were selected as being the best models according to the statistical analysis they conducted [9]. Nonetheless, these authors also conclude that most of the models tested could be used to fit the growth profile. 

The Baranyi and Roberts and Gompertz-m models yielded higher estimates for the duration of the lag phase at higher initial glucose concentrations. Despite their similar trends along the three glucose concentrations, the Baranyi and Roberts model tended to provide slightly lower parameter values compared to the Gompertz-m model. Conversely, the Buchanan model’s estimates for the lag phase duration are consistently lower than those of the other models, although this trend is not consistent across experimental conditions with distinct initial glucose concentrations. 

When it comes to the growth rate, the three mathematical models demonstrated a similar response pattern with respect to the initial glucose concentration, with the Baranyi and Roberts and Gompertz-m models showing the closest similarity. Conversely, the Buchanan model yielded slightly lower maximum specific growth rate values across all initial glucose concentrations. The maximum specific growth rate was slightly higher in the medium containing 200 g/L of glucose, followed by that with 150 g/L of glucose, and ultimately the medium with 250 g/L of glucose. The significant differences observed in mean maximum specific growth rate values were primarily limited to the media with 200 g/L and 250 g/L of glucose. This suggests that the strain used is well adapted for growth in media with 150 g/L and 200 g/L of glucose, while a glucose concentration of 250 g/L presents a significant growth barrier.

It is important to note that more replications would enhance confidence in these findings. 

Regarding the final viable population, all three models exhibited a comparable behavior as well. Higher counts were produced by the medium containing 250 g/L of glucose, followed by counts obtained with 200 g/L glucose, and finally with 150 g/L of glucose. Despite the noted significant variations in average viable population values across glucose concentrations, from an enological perspective, these viable populations remained suitable to carry on efficient alcoholic fermentations. Focusing on the parameter estimates, as observed with the lag and maximum specific growth rate, the Buchanan model yielded lower average estimates compared to those obtained using the Baranyi and Roberts and Gompertz-m models (with the Gompertz-m producing the highest values).

Buchanan’s model may be less effective in culture kinetics with smooth transitions between growth phases, taking into account the nature of the mathematical function involved in its definition (continuous function but not differentiable in the transition point from one straight line to the other), potentially contributing to lower values for the two main kinetics parameters (lag and mumax). The same authors that derived the model [11] tested it using growth data for *Escherichia coli* and compared it against Buchanan’s and Baranyi and Roberts models. They suggested that the linear model was shown to be more robust than the others, especially if the available data were minimal, which is probably related to its simplicity. While the model performs well with abrupt changes, making it a straightforward choice for modelling microbial growth, given its apparent simplicity—its three linear segments can be determined subjectively without dedicated tools—it is crucial to note that achieving objectivity in results requires the application of (non-linear) optimization algorithms based on defined criteria. 

All in all, despite differences observed in the average values of the parameters (Table 4), all three mathematical models exhibit good fits with the kinetics observed across the various glucose concentrations (good visual inspections and analysis of residuals) and enable accurate parameter estimation for growth analysis (small residual standard errors). Nonetheless, as we have shown, different models can lead to varying and even significantly different estimates for the same growth parameters. This strongly suggests that specifying the model employed for parameter estimation is crucial to ensure meaningful inter-study comparisons, between diverse experimental works. In our study, the lag phase and growth rate parameters have been particularly sensitive to this.

Computation of the correlations between model parameters obtained for each of the three models has allowed us to explore the potential impact of the selected model on the study outcomes. This analysis highlighted that the relationship among the estimated kinetic values obtained from the alternative models exhibited distinct behaviors. Notably, it becomes evident that the nature of the relationship, its strength, or its absence, varies depending on which model is applied to the same dataset. 

Furthermore, the study introduced confidence interval estimates for these kinetic parameters (Table 3), a strategic approach that provides insights into the potential range of the real parameter values. This not only enhances the comparability of values across studies but also enables more precise conclusions regarding observed growth kinetics. By encompassing inherent data variability and model fitting adequacy, these intervals contribute to a comprehensive understanding of experimental outcomes. 

## 5. Conclusions

We conclude that both Pre I and Pre II protocols provided pre-cultures with similar viability and growth. This suggests that short protocols such as Pre I may represent a more cost-effective approach. Moreover, our study highlights the need of using a high inoculation rate to slow down dissolved oxygen depletion in the media and to encourage glucose to be predominantly processed towards the fermentative metabolism of *S. cerevisiae* at early fermentative steps. 

We have assessed the performance of three well-known predictive primary growth models: the Buchanan three-phase linear model, the modified Gompertz model, and the Baranyi and Roberts model. The assessment has not highlighted any model as privileged and the selection may depend on the specific interest or characteristics of the study at hand and the available modelling knowledge. In that respect, the use of available software, such as the one used in this contribution, can help to fulfill modelling knowledge gaps, allowing a deeper focus on the microbiological work. 

The analysis conducted also provided evidence that the relationship between kinetic parameters is not fixed but depends on the model used. This hinders inter-study comparisons and stresses the need of using standardized, well-defined growth models in microbiological studies. From this vantage point, we strongly advocate for generalized use and reporting of the confidence intervals of the kinetic parameters to further facilitate objective inter-study comparisons.

## Figures and Tables

**Table 1:** Mean and standard deviation (SD) for the viable population (expressed as Log CFU/mL) and the percentage of living budding cells (%) at the end of the pre-cultures prepared using Pre I and Pre II protocols for the three initial glucose concentrations (150 g/L, 200 g/L, and 250 g/L). The means of viable population were subjected to a separation analysis utilizing interactions between two factors: protocol and initial glucose concentration. This analysis was conducted using the Fisher Least Significant Difference method. Means that do not share a letter are statistically different.

Protocol	Initial Glucose (g/L)	Viable Population (Log CFU/mL)		Living Budding Cells (%)	
		Mean	SD	Mean	SD
Pre I	150	6.26 b	0.24	25.1 a	7.5
	200	6.81 a	0.13	24.5 a	2.5
	250	6.94 a	0.30	18.1 a	12.1
Pre II	150	6.87 a	0.19	35.8 a	4.3
	200	6.77 a	0.13	29.0 a	13.7
	250	6.64 a	0.11	27.2 a	14.3

**Table 2:** Means and standard deviations (SDs) of the initial population and biomass increase at 48 h ((Final population at 48 h (Log CFU/mL)) − Initial population (Log CFU/mL)) of growth cultures with distinct initial glucose concentrations and using inocula produced following protocols Pre I and Pre II. Analyzed data are based on optical densities at 600 nm that have been converted to CFU/mL units using a calibration procedure.

Protocol	Initial Glucose (g/L)	Initial Population (Log CFU/mL)		Biomass Increase at 48 h (Log CFU/mL)	
		Mean	SD	Mean	SD
Pre I	150	5.13	0.03	1.67	0.06
	200	5.13	0.04	1.48	0.05
	250	5.14	0.03	1.43	0.13
Pre II	150	5.11	0.01	1.46	0.17
	200	5.13	0.03	1.51	0.09
	250	5.14	0.01	1.32	0.09

**Table 3:** Mean and confidence interval of the parameter’s values estimated with the models of Baranyi and Roberts, Buchanan, and Gompertz-m using data from *S. cerevisiae* cultures growing in three initial glucose concentrations (150 g/L, 200 g/L, and 250 g/L). Four model parameters encompassing initial population size (LOG10N0), lag phase duration (lag), maximum specific growth rate (mumax), and final population size (LOG10Nmax) were estimated. The “95% CI” annotation denotes the confidence interval associated with a 95% confidence level.

Fitted Model	Initial Glucose (g/L)	LOG10N0 (Log CFU/mL)	lag (h)	mumax (h^−1^)	LOG10Nmax (Log CFU/mL)
		Mean 95% CI	Mean 95% CI	Mean 95% CI	Mean 95% CI
Buchanan					
	150	4.54 (4.48, 4.60)	4.7 (3.41, 5.93)	0.50 (0.23, 0.77)	7.15 (7.04, 7.26)
	200	4.50 (4.16, 4.84)	5.5 (4.70, 6.38)	0.52 (0.26, 0.79)	7.22 (7.06, 7.38)
	250	4.53 (3.74, 5.32)	4.8 (2.70, 6.92)	0.37 (0.19, 0.55)	7.25 (7.17, 7.34)
Gompertz-m					
	150	4.51 (4.40, 4.62)	5.3 (4.10, 6.54)	0.60 (0.33, 0.87)	7.21 (7.08, 7.33)
	200	4.49 (4.19, 4.79)	6.6 (5.67, 7.47)	0.70 (0.34, 1.05)	7.26 (7.07, 7.45)
	250	4.60 (3.83, 5.36)	7.1 (3.11, 11.10)	0.50 (0.29, 0.72)	7.31 (7.20, 7.43)
Baranyi and Roberts					
	150	4.43 (4.25, 4.60)	5.0 (4.25, 4.60)	0.59 (0.19, 0.98)	7.17 (7.05, 7.30)
	200	4.43 (4.14, 4.73)	6.5 (5.82, 7.14)	0.67 (0.34, 1.00)	7.22 (7.06, 7.39)
	250	4.51 (3.76, 5.25)	6.6 (2.91, 10.32)	0.47 (0.31, 0.64)	7.27 (7.19, 7.36)

**Table 4:** *p*-values of the ANOVA tests contrasting the linear model with interaction of the estimated values of the kinetic parameters corresponding to the duration of the lag phase, the specific growth rate, and the initial and final population, for the two factors, the growth model fitted to the data (Model) (Baranyi and Roberts, Buchanan, Gompertz-m) and the initial amount of glucose in the medium (initial glucose) (150, 200, 250 g/L).

Source of Variation	Initial Population (LOG10N0)	Duration of the Lag Phase (lag)	Specific Growth Rate (mumax)	Final Population (LOG10Nmax)
Initial glucose	0.739	0.017 *	0.012 *	0.003 **
Model	0.661	0.016 *	0.048 *	0.132
Initial glucose * Model	0.991	0.543	0.989	0.997

* *p*-value < 0.05; ** *p*-value < 0.01.

**Table 5:** Pearson correlations and *p*-values of the comparisons between pairs of kinetic parameters estimated from distinct models. In the table, *p*-values are found in brackets. Scatter diagrams can be found in the Supplementary Material (Figure S6).

	Baranyi and Roberts			Buchanan			Gompertz-m		
	lag	mumax	LOG10N0	lag	mumax	LOG10N0	lag	mumax	LOG10N0
mumax	0.285 (0.457)			0.707 (0.033)			0.121 (0.757)		
LOG10N0	−0.078 (0.843)	−0.382 (0.311)		−0.757 (0.018)	−0.473 (0.199)		−0.158 (0.686)	−0.627 (0.070)	
LOG10Nmax	0.302 (0.429)	−0.642 (0.062)	0.037 (0.924)	0.092 (0.814)	−0.606 (0.083)	−0.069 (0.860)	0.475 (0.197)	−0.553 (0.123)	0.072 (0.853)

## Data Availability

The original contributions presented in the study are included in the article/Supplementary Material; further inquiries can be directed to the corresponding author.

## Supplementary Materials

The following supporting information can be downloaded at: https://www.mdpi.com/article/10.3390/microorganisms12071449/s1, Figure S1: Calibration data. Figure S2: Viable cell count (Log CFU/mL) and percentages of viable cells in budding obtained from the two protocols (Pre I and Pre II) at the three glucose concentrations (150, 200 and 250 g/L). In the figure, values of the three replicates (circle) and their means (diamond) are depicted. Figure S3: Fits of the three growth models applied (Baranyi & Roberts, Buchanan, Gompertz-m) to the different sets of experimental data. Repetitions for each initial glucose concentration used (150, 200, 250 g/L) are denoted using capital letters (A, B, C). Table S1: Point estimates and corresponding confidence intervals for the three growth models (Baranyi & Roberts, Buchanan, Gompertz-m) fitted to the different sets of experimental data obtained at three initial glucose concentration used (150, 200, 250 g/L). Replications are denoted using capital letters (A, B, and C). Figure S4: Residual standard error of the three growth models (Baranyi & Roberts, Buchanan, and Gompertz-m) considering (A) or disregarding (B) the initial glucose concentration of the cultures. Figure S5: Interaction plots of the of the ANOVA analysis conducted over the kinetic parameters estimated with the three models (Barany&Roberts, Buchanan, and Gompertz-m) on three initial glucose concentrations (150, 200 and 250 g/L). Kinetic parameters estimated are duration of the lag phase (lag), maximum specific growth rate (mumax) and maximum population density (LOG10Nmax), plots A, B and C, respectively. Figure S6: Scatter diagrams for the estimates obtained for the kinetic parameters, duration of the adaptation or lag phase (lag), maximum specific growth rate (mumax), and initial population and final population (LOG10N0 and LOG10Nmax, respectively), according to the model used (Baranyi & Roberts, Buchanan, Gompertz-m). The three initial glucose concentrations of 150 (blue circles), 200 (red squares), and 250 g/L (green diamonds) are shown. Table S2: The table includes: (i) Pearson correlations and *p*-values (in brackets); (ii) linear regression lines to show the relationship between the estimates of the four kinetic parameters studied (duration of the lag phase (lag), maximum specific growth rate (mumax), initial population density (LOG10N0), and final population density (LOG10Nmax)) obtained with the three models used; (iii) significance (*p*-value) of the contrast for a value equal to or different from 0 of the ordinate at the origin of the regression line, and (iv) the confidence interval for the estimated intercept and the estimated slope of the regression line. Bu: Buchanan model, Gom: Gompertz-m model, B&R: Baranyi & Roberts model.

## References

[1] Ciani M., Comitini F., Mannazzu I., Domizio P. (2010). Controlled Mixed Culture Fermentation: A New Perspective on the Use of Non-*Saccharomyces* Yeasts in Winemaking. FEMS Yeast Res..

[2] Barbosa C., Ramalhosa E., Vasconcelos I., Reis M., Mendes-Ferreira A. (2022). Machine Learning Techniques Disclose the Combined Effect of Fermentation Conditions on Yeast Mixed-Culture Dynamics and Wine Quality. Microorganisms.

[3] Ganucci D., Guerrini S., Mangani S., Vicenzini M., Granchi L. (2018). Quantifying the Effects of Ethanol and Temperature on the Fitness Advantage of Predominant *Saccharomyces cerevisiae* Strains Occurring in Spontaneous Wine Fermentations. Front. Microbiol..

[4] Guerrini S., Barbato D., Guerrini L., Mari E., Buscioni G., Mangani S., Romboli Y., Galli V., Parenti A., Granchi L. (2021). Selection of Indigenous *Saccharomyces cerevisiae* Strains and Exploitation of a Pilot-Plant to Produce Fresh Yeast Starter Cultures in a Winery. Fermentation.

[5] Kontkanen D., Inglis D.L., Pickering G.J., Reynolds A. (2004). Effect of yeast inoculation rate; acclimatization; and nutrient addition on icewin fermentation. Am. J. Enol. Vitic..

[6] Arroyo-López F.N., Orlić S., Querol A., Barrio E. (2009). Effects of temperature; pH and sugar concentration on the growth parameters of *Saccharomyces cerevisiae*; *S. kudriavzevii* and their interspecific hybrid. Int. J. Food Microbiol..

[7] Yi-Huang C., Ku-Shang C., Chien-Yu C., Chuan-Liang H., Tsan-Chang C., Hung-Der J. (2018). Enhancement of the efficiency of bioethanol production by *Saccharomyces cerevisiae* via gradually batch-wise and fed-batch increasing the glucose concentration. Fermentation.

[8] Miller K.V., Block D.E. (2020). A review of wine fermentation process modelling. J. Food Eng..

[9] Halmi M.I.E., Shukor M.S., Johari W.L.W., Shukor M.Y. (2014). Evaluation of several mathematical models for fitting the growth of the algae *Dunaliella tertiolecta*. Asian J. Plant Biol..

[10] Portell X., Gras A., Ginovart M. (2014). INDISIM-Saccha, an individual-based model to tackle Saccharomyces cerevisiae fermentations. Ecol. Model..

[11] Buchanan R.L., Whiting R.C., Damert W.C. (1997). When is simple good enough: A comparison of the Gompertz, Baranyi and three phase linear models for fitting bacterial growth curves. Food Microbiol..

[12] Baranyi J., Roberts T.A. (1994). A dynamic approach to predicting bacterial growth in food. Int. J. Food Microbiol..

[13] Gibson A.M., Bratchell N., Roberts T.A. (1988). Predicting microbial growth: Growth responses of salmonellae in a laboratory medium as affected by pH; sodium chloride and storage temperature. Int. J. Food Microbiol..

[14] Zwietering M.H., Jongenburger I., Rombouts F.M., van ’t Riet K. (1990). Modeling of the Bacterial Growth Curve. Appl. Environ. Microbiol..

[15] McKellar R.C. (1997). A heterogeneous population model for the analysis of bacterial growth kinetics. Int. J. Food Microbiol..

[16] López S., Prieto M., Dijkstra J., Dhanoa M.S., France J. (2004). Statistical evaluation of mathematical models for microbial growth. Int. J. Food Microbiol..

[17] D’Amato D., Corbo M.R., Nobile M.A.D., Sinigaglia M. (2006). Effects of temperature, ammonium and glucose concentrations on yeast growth in a model wine system. Int. J. Food Sci. Technol..

[18] R Core Team (2019). R: A Language and Environment for Statistical Computing.

[19] Baty F., Ritz C., Charles S., Brutsche M., Flandrois J.P., Delignette-Muller M.L. (2015). A Toolbox for Nonlinear Regression in R: The Package nlstools. J. Stat. Soft..

[20] Baty F., Delignette-Muller M.L. nlsMicrobio: Data Sets and Nonlinear Regression Models Dedicated to Predictive Microbiology; R Package Version 0.0-1; 2015. https://github.com/lbbe-software/nlsMicrobio.

[21] Minitab Inc. (2000). Minitab Statistical Software Version 17.2.1.

[22] Carbó R., Ginovart M., Carta A., Portell X., Del Valle L.J. (2015). Effect of aerobic and microaerophilic culture in the growth dynamics of *Saccharomyces cerevisiae* and in training of quiescent and non-quiescent subpopulations. Arch. Microbiol..

[23] Coelho M.A.Z., Belo I., Pinheiro R., Amaral A.I., Mota M., Coutinho J.A.P., Ferreira E.C. (2004). Effect of hyperbaric stress on yeast morphology: Study by automated image analysis. Appl. Microbiol. Biotechnol..

[24] Brejning J., Jespersen L. (2002). Protein expression during lag phase and growth initiation in *Saccharomyces cerevisiae*. Int. J. Food Microbiol..

[25] Vázquez-Lima F., Silva P., Barreiro A., Martínez-Moreno R., Morales P., Quirós M., González R., Albiol J., Ferrer P. (2014). Use of chemostat cultures mimicking different phases of wine fermentations as a tool for quantitative physiological analysis. Microb. Cell Fact..

[26] Vigentini I., Fabrizio V., Faccincani M., Picozzi C., Comasio A., Foschino R. (2014). Dynamics of *Saccharomyces cerevisiae* populations in controlled and spontaneous fermentations for Franciacorta D.O.C.G. base wine production. Ann. Microbiol..

[27] Laverty D.J., Kury A.L., Kuksin D., Pirani A., Flanagan K., Li-Ying Chan L. (2013). Automated quantification of budding *Saccharomyces* using a novel image cytometry method. J. Ind. Microbiol. Biotechnol..

[28] Portell X., Ginovart M., Carbó R., Gras A., Vives-Rego J. (2011). Population analysis of a commercial *Saccharomyces cerevisiae* wine yeast in a batch culture by electric particle analysis; light diffraction and flow cytometry. FEMS Yeast Res..

[29] Bisschops M.M., Vos T., Martínez-Moreno R., De la Torre Cortés P., Daran-Lapujade P.D. (2015). Oxygen availability strongly affects chronological lifespan and thermotolerance in batch cultures of *Saccharomyces cerevisiae*. Microb. Cell.

[30] Gómez-Pastor R., Pérez-Torrado R., Garre E., Matallana E., Matovic D. (2011). Recent Advances in Yeast Biomassa Production. Biomass—Detection; Production and Usage.

[31] Kemp B., Plante J., Inglis D.L. (2020). Nutrient addition to low pH base wines (L. cv. Riesling) during yeast acclimatization for sparkling wine: Its influence on yeast cell growth; sugar consumption and nitrogen usage. Beverages.

[32] Ginovart M., Carbó R., Blanco M., Portell X. (2018). Digital image analysis of yeasts single cells growing in two different oxygen concentrations to analyze the population growth and to assist Individual-Based Modeling. Front. Microbiol..

[33] Marbà-Ardébol A., Emmerich J., Muthig M., Neubauer P., Junne S. (2018). Real-time monitoring of the budding index in *Saccharomyces cerevisiae* batch cultivation with in situ microscopy. Microb. Cell Fact..

[34] Minebois R., Pérez-Torrado R., Querol A. (2020). A time course metabolism comparison among *Saccharomyces uvarum* and *S. kudriavzevii* species in wine fermentation. Food Microbiol..

[35] Alonso-del-Real J., Contreras-Ruiz A., Castiglioni G.L., Barrio E., Querol A. (2017). The use of mixed populations of *Saccharomyces cerevisiae* and *S. kudriavzevii* to reduce ethanol content in wine: Limited aeration; inoculum proportions; and sequential inoculation. Front. Microbiol..

[36] Heyland J., Fu J., Blank L.M. (2009). Correlation between TCA cycle flux and glucose uptake rate during respiro-fermentative growth of *Saccharomyces cerevisiae*. Microbiology.

[37] Tilloy V., Ortiz-Julien A., Dequin S. (2014). Reduction of ethanol yield and improvement of glycerol formation by adaptive evolution of the wine yeast *Saccharomyces cerevisiae* under hyperosmotic conditions. Appl. Environ. Microbiol..

[38] Walker G.M., Stewart G.G. (2016). *Saccharomyces cerevisiae* in the production of fermented beverages. Beverages.

[39] Schwinn M., Durner D., Delgado A., Fischer U. (2019). Distribution of yeast cells; temperature; and fermentation By-products in white wine fermentations. Am. J. Enol. Vitic..

[40] Marsit S., Dequin S. (2015). Diversity and adaptive evolution of *Saccharomyces* wine yeast: A review. FEMS Yeast Res..

