# Supplementary material for: Adaptation of Saccharomyces to High Glucose Concentrations and Its Impact on Growth Kinetics of Alcoholic Fermentations
Source: Microorganisms. 2024 Jul 17;12(7):1449. doi: 10.3390/microorganisms12071449 (PMC11278885; doi:10.3390/microorganisms12071449)
Supplement: Supplementary file 1 [file microorganisms-12-01449-s001.zip › microorganisms-3083543-supplementary.pdf]

Supplementary Material

Adaptation of *Saccharomyces* to High Glucose Concentrations and Its Impact on Growth Kinetics of Alcoholic Fermentations

Marta Ginovart[1], Rosa Carbó[2], Xavier Portell[3,*]

Author affiliation(s)

[1] Departament de Matemàtiques, Universitat Politècnica de Catalunya-BarcelonaTECH, 08860 Castelldefels, Catalunya, Spain; marta.ginovart@upc.edu

[2] Escola d'Enginyeria Agroalimentària i de Biosistemes de Barcelona, Universitat Politècnica de Catalunya-BarcelonaTECH, 08860 Castelldefels, Catalunya, Spain; rosa.carbo@upc.edu

[3] Departamento de Ciencias Agrarias y del Medio Natural, Escuela Politécnica Superior de Huesca, Universidad de Zaragoza, Ctra. Cuarte s/n, 22071 Huesca, Aragón, Spain

*Correspondence: xportell@unizar.es or xavier.portell.canal@gmail.com

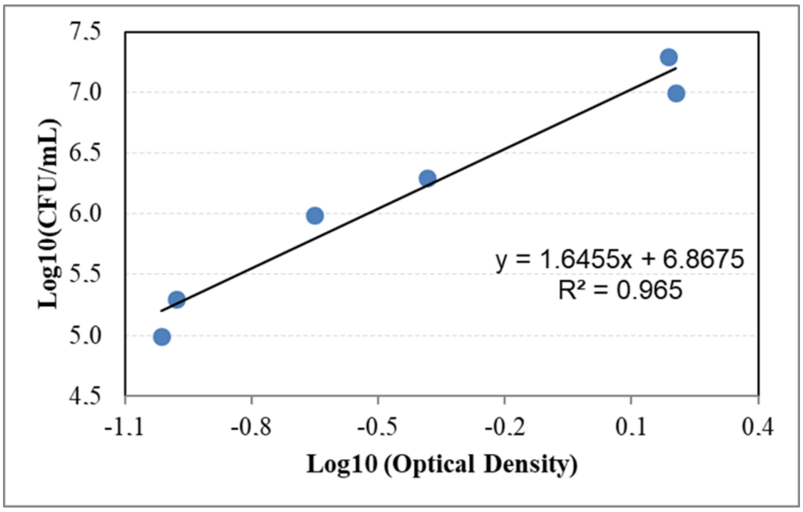

Figure S1. Calibration curve to transform OD values at 600 nm to CFU mL^{-1}. A logarithmic transformation was performed for both the OD values and the cell counts to equalize the differences between the data points.

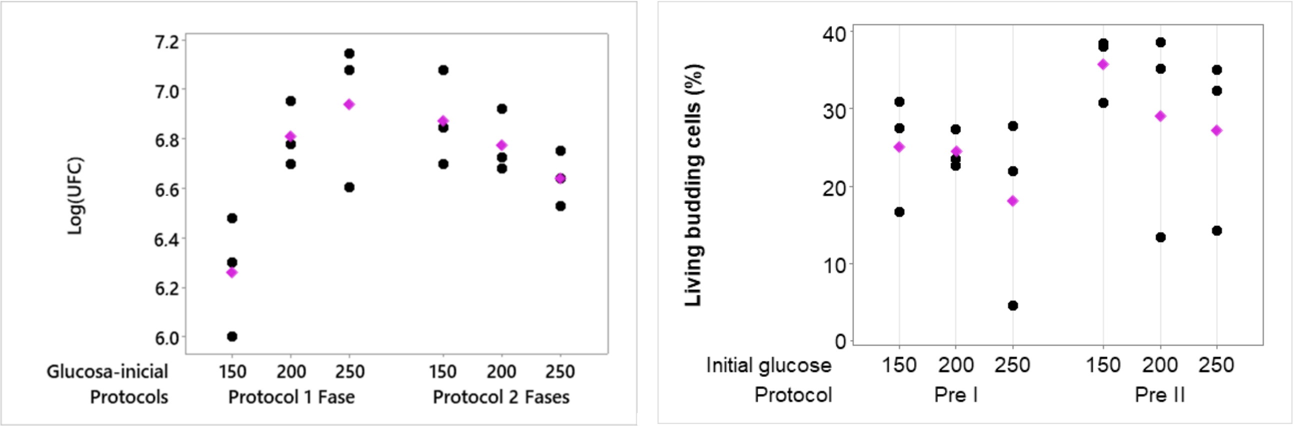

Figure S2. Viable cell count (Log CFU/mL) and percentages of viable cells in budding obtained from the two protocols (Pre I and Pre II) at the three glucose concentrations (150, 200 and 250 g/L). In the figure, values of the three replicates (circle) and their means (diamond) are depicted.

	Baranyi&Roberts	Buchanan	Gompertz-m
150-A	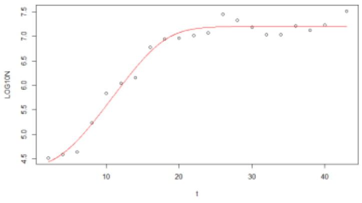	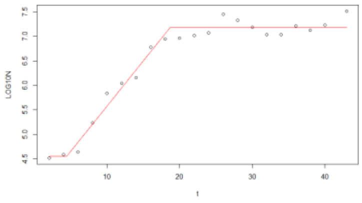	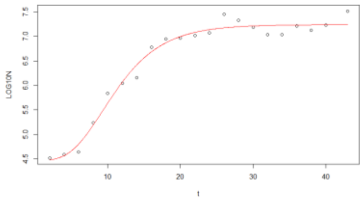
150-B	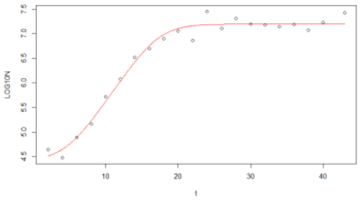	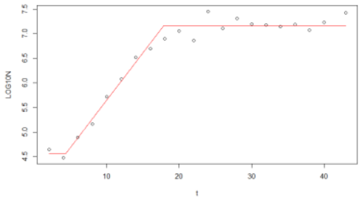	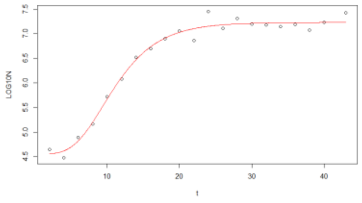
150-C	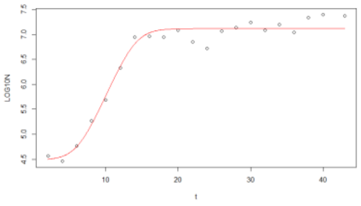	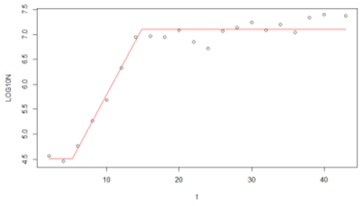	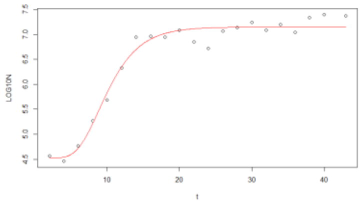
200-A	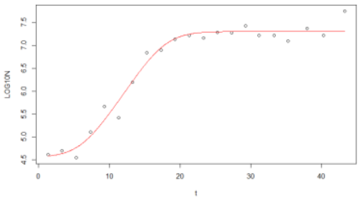	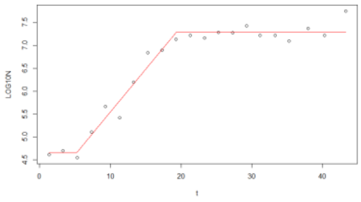	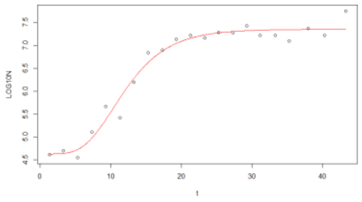
200-B	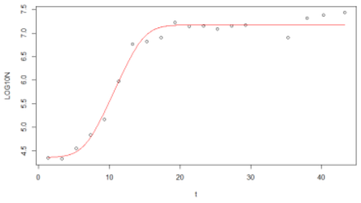	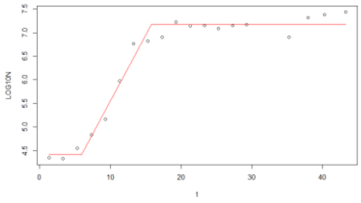	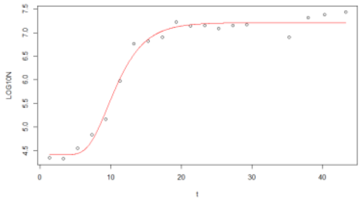
200-C	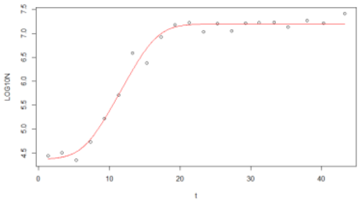	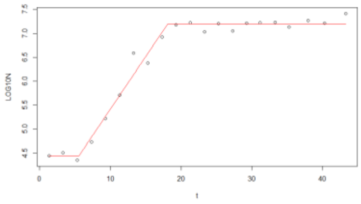	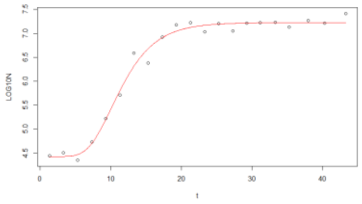
250-A	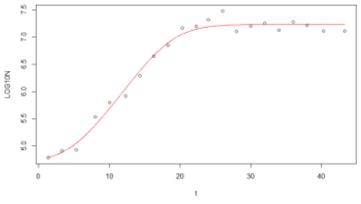	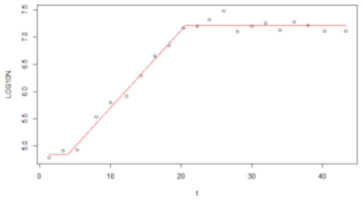	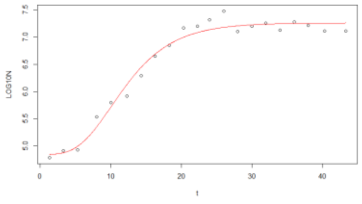

250-B	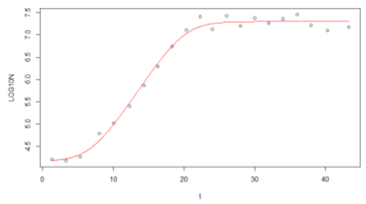	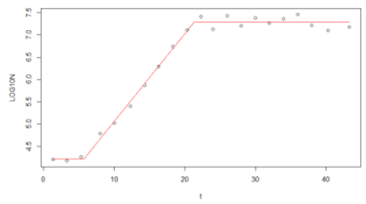	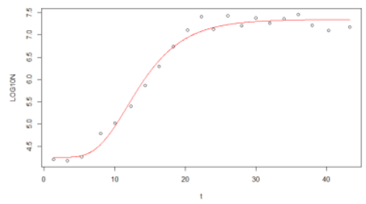
250-C	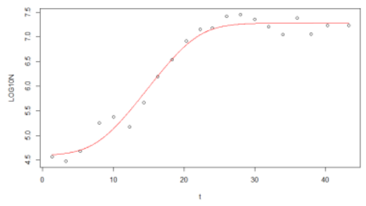	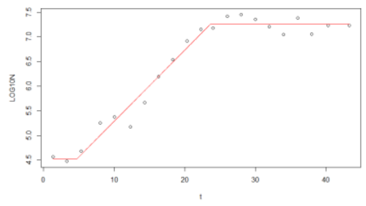	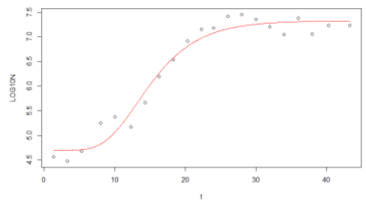

Figure S3. Fits of the three growth models applied (Baranyi&Roberts, Buchanan, Gompertz-m) to the different sets of experimental data. Repetitions for each initial glucose concentration used (150, 200, 250 g/L) are denoted using capital letters (A, B, C).

Table S1. Point estimates, and corresponding confidence intervals, of the four kinetic parameters for the three growth models (Baranyi&Roberts, Buchanan, Gompertz-m) fitted to the different sets of experimental data obtained at three initial glucose concentration used (150, 200, 250 g/L). Replications are denoted using capital letters (A, B, and C). The identification of the parameters is as follows: the duration of the adaptation phase (lag), maximum specific growth rate (mumax), initial population (LOG10N0), and carrying capacity or final population (LOG10Nmax). Reparametrized formulas of the growth models (used by nlsMicrobio R package) are introduced at the beginning of the table.

	Baranyi&Roberts	Buchanan	Gompertz-m
	$LOG10N := t \mapsto LOG10Nmax + \log10\left(\left(-1 + e^{mumax \cdot lag} + e^{mumax \cdot t}\right) / \left(e^{mumax \cdot t} - 1 + e^{mumax \cdot lag} \cdot 10^{LOG10Nmax - LOG10N0}\right)\right)$	$LOG10N := t \mapsto LOG10N0 + \frac{1}{\log(10)}\left((lag \le t) \cdot \left(t \le lag + \frac{(LOG10Nmax - LOG10N0) \cdot \log(10)}{mumax}\right) \cdot mumax \cdot (t - lag)\right) + (lag \le t) \cdot \left(lag + \frac{(LOG10Nmax - LOG10N0) \cdot \log(10)}{mumax} < t\right) \cdot (LOG10Nmax - LOG10N0)$	$LOG10N := t \mapsto LOG10N0 + (LOG10Nmax - LOG10N0) \cdot e^{-e^{\frac{mumax \cdot e \cdot (lag - t)}{(LOG10Nmax - LOG10N0) \cdot \log(10)} + 1}}$
150-A	Parameters:<br>Estimate Std. Error t value Pr(>|t|)<br>lag 4.07882 1.79188 2.276 0.0361<br>mumax 0.48015 0.05781 8.306 2.18e-07<br>LOG10N0 4.35091 0.20271 21.464 9.41e-14<br>LOG10Nmax 7.20669 0.05070 142.150 < 2e-16<br><br>t-based confidence interval:<br>2.5% 97.5%<br>lag 0.2982701 7.8593602<br>mumax 0.3581823 0.6021258<br>LOG10N0 3.9232365 4.7785912<br>LOG10Nmax 7.0997277 7.3136537	Parameters:<br>Estimate Std. Error t value Pr(>|t|)<br>lag 4.43597 1.00476 4.415 0.000379<br>mumax 0.42479 0.03755 11.313 2.46e-09<br>LOG10N0 4.55500 0.12203 37.328 < 2e-16<br>LOG10Nmax 7.18325 0.04982 144.192 < 2e-16<br><br>t-based confidence interval:<br>2.5% 97.5%<br>lag 2.3161067 6.5558323<br>mumax 0.3455682 0.5040035<br>LOG10N0 4.2975456 4.8124545<br>LOG10Nmax 7.0781447 7.2883553	Parameters:<br>Estimate Std. Error t value Pr(>|t|)<br>lag 4.84104 1.08919 4.445 0.000355<br>mumax 0.52633 0.05492 9.584 2.88e-08<br>LOG10N0 4.46483 0.14852 30.063 3.53e-16<br>LOG10Nmax 7.24196 0.05551 130.469 < 2e-16<br><br>t-based confidence interval:<br>2.5% 97.5%<br>lag 2.5430593 7.1390191<br>mumax 0.4104628 0.6422051<br>LOG10N0 4.1514929 4.7781745<br>LOG10Nmax 7.1248538 7.3590732
150-B	Parameters:<br>Estimate Std. Error t value Pr(>|t|)<br>lag 4.75809 1.36644 3.482 0.00285<br>mumax 0.51112 0.05543 9.220 5.02e-08<br>LOG10N0 4.44905 0.15517 28.673 7.77e-16<br>LOG10Nmax 7.19698 0.04254 169.191 < 2e-16<br><br>t-based confidence interval:<br>2.5% 97.5%<br>lag 1.8751630 7.6410231<br>mumax 0.3941663 0.6280738<br>LOG10N0 4.1216753 4.7764250<br>LOG10Nmax 7.1072377 7.2867307	Parameters:<br>Estimate Std. Error t value Pr(>|t|)<br>lag 4.31519 0.92945 4.643 0.000233<br>mumax 0.44250 0.04303 10.283 1.03e-08<br>LOG10N0 4.56029 0.11056 41.247 < 2e-16<br>LOG10Nmax 7.16483 0.04336 165.221 < 2e-16<br><br>t-based confidence interval:<br>2.5% 97.5%<br>lag 2.3542188 6.2761666<br>mumax 0.3517125 0.5332859<br>LOG10N0 4.3270268 4.7935471<br>LOG10Nmax 7.0733365 7.2563208	Parameters:<br>Estimate Std. Error t value Pr(>|t|)<br>lag 5.28846 0.81083 6.522 5.22e-06<br>mumax 0.54767 0.04945 11.076 3.39e-09<br>LOG10N0 4.55075 0.10872 41.858 < 2e-16<br>LOG10Nmax 7.23015 0.04357 165.955 < 2e-16<br><br>t-based confidence interval:<br>2.5% 97.5%<br>lag 3.5777624 6.9991622<br>mumax 0.4433468 0.6519901<br>LOG10N0 4.3213761 4.7801304<br>LOG10Nmax 7.1382325 7.3220690
150-C	Parameters:<br>Estimate Std. Error t value Pr(>|t|)<br>lag 6.17206 1.03137 5.984 1.48e-05<br>mumax 0.77019 0.12085 6.373 6.94e-06<br>LOG10N0 4.48810 0.14523 30.903 2.23e-16<br>LOG10Nmax 7.11467 0.04853 146.606 < 2e-16<br><br>t-based confidence interval:<br>2.5% 97.5%<br>lag 3.9960588 8.348067<br>mumax 0.5152306 1.025159<br>LOG10N0 4.1816906 4.794517<br>LOG10Nmax 7.0122847 7.217060	Parameters:<br>Estimate Std. Error t value Pr(>|t|)<br>lag 5.24793 0.73522 7.138 1.66e-06<br>mumax 0.62220 0.06432 9.673 2.51e-08<br>LOG10N0 4.51530 0.12492 36.144 < 2e-16<br>LOG10Nmax 7.10125 0.04722 150.395 < 2e-16<br><br>t-based confidence interval:<br>2.5% 97.5%<br>lag 3.6967549 6.7991142<br>mumax 0.4864966 0.7579056<br>LOG10N0 4.2517314 4.7788686<br>LOG10Nmax 7.0016290 7.2008681	Parameters:<br>Estimate Std. Error t value Pr(>|t|)<br>lag 5.82068 0.82944 7.018 2.07e-06<br>mumax 0.72639 0.10287 7.061 1.91e-06<br>LOG10N0 4.52379 0.12856 35.187 < 2e-16<br>LOG10Nmax 7.14765 0.05262 135.825 < 2e-16<br><br>t-based confidence interval:<br>2.5% 97.5%<br>lag 4.0707092 7.5706468<br>mumax 0.5093493 0.9434372<br>LOG10N0 4.2525480 4.7950413<br>LOG10Nmax 7.0366221 7.2586753
200-A	Parameters:<br>Estimate Std. Error t value Pr(>|t|)<br>lag 6.17532 1.41822 4.354 0.000432<br>mumax 0.54309 0.07660 7.090 1.81e-06<br>LOG10N0 4.56977 0.15094 30.276 3.13e-16<br>LOG10Nmax 7.30170 0.05939 122.935 < 2e-16<br><br>t-based confidence interval:<br>2.5% 97.5%<br>lag 3.1831270 9.167510<br>mumax 0.3814755 0.704709<br>LOG10N0 4.2513272 4.888221<br>LOG10Nmax 7.1763921 7.427016	Parameters:<br>Estimate Std. Error t value Pr(>|t|)<br>lag 5.22600 1.00219 5.215 7.01e-05<br>mumax 0.42897 0.03453 12.424 5.90e-10<br>LOG10N0 4.65804 0.13743 33.894 < 2e-16<br>LOG10Nmax 7.29227 0.05860 124.441 < 2e-16<br><br>t-based confidence interval:<br>2.5% 97.5%<br>lag 3.1115598 7.3404448<br>mumax 0.3561284 0.5018194<br>LOG10N0 4.3680852 4.9479901<br>LOG10Nmax 7.1686379 7.4159102	Parameters:<br>Estimate Std. Error t value Pr(>|t|)<br>lag 6.14979 1.11692 5.506 3.86e-05<br>mumax 0.54649 0.07450 7.335 1.17e-06<br>LOG10N0 4.62949 0.13458 34.401 < 2e-16<br>LOG10Nmax 7.34897 0.07046 104.304 < 2e-16<br><br>t-based confidence interval:<br>2.5% 97.5%<br>lag 3.7932976 8.5062920<br>mumax 0.3893003 0.7036765<br>LOG10N0 4.3455629 4.9134205<br>LOG10Nmax 7.2003197 7.4976247
200-B	Parameters:<br>Estimate Std. Error t value Pr(>|t|)<br>lag 6.67856 0.77007 8.673 3.14e-07<br>mumax 0.80908 0.10053 8.048 8.00e-07	Parameters:<br>Estimate Std. Error t value Pr(>|t|)<br>lag 5.89952 0.72953 8.087 7.54e-07<br>mumax 0.64142 0.06585 9.741 7.05e-08	Parameters:<br>Estimate Std. Error t value Pr(>|t|)<br>lag 6.77742 0.60138 11.27 1.01e-08<br>mumax 0.83561 0.10092 8.28 5.62e-07

<table>
<tr><td></td><td>LOG10N0 4.35894 0.10734 40.610 < 2e-16
LOG10Nmax 7.17568 0.04784 150.002 < 2e-16

t-based confidence interval:
2.5% 97.5%
lag 5.0371865 8.319934
mumax 0.5948045 1.023354
LOG10N0 4.1301557 4.587715
LOG10Nmax 7.0737192 7.277645</td><td>LOG10N0 4.41162 0.10442 42.247 < 2e-16
LOG10Nmax 7.17155 0.05453 131.506 < 2e-16

t-based confidence interval:
2.5% 97.5%
lag 4.3445627 7.4544803
mumax 0.5010707 0.7817791
LOG10N0 4.1890400 4.6341928
LOG10Nmax 7.0553167 7.2877901</td><td>LOG10N0 4.41349 0.09260 47.66 < 2e-16
LOG10Nmax 7.21167 0.05126 140.69 < 2e-16

t-based confidence interval:
2.5% 97.5%
lag 5.4956005 8.059243
mumax 0.6204996 1.050715
LOG10N0 4.2161115 4.610872
LOG10Nmax 7.1024137 7.320930</td></tr>
<tr><td>200-C</td><td>Parameters:
Estimate Std. Error t value Pr(>|t|)
lag 6.58142 0.88877 7.405 1.03e-06
mumax 0.65357 0.07137 9.158 5.53e-08
LOG10N0 4.37279 0.10777 40.574 < 2e-16
LOG10Nmax 7.19766 0.04404 163.427 < 2e-16

t-based confidence interval:
2.5% 97.5%
lag 4.7062704 8.456563
mumax 0.5029933 0.804140
LOG10N0 4.1454036 4.600169
LOG10Nmax 7.1047420 7.290583</td><td>Parameters:
Estimate Std. Error t value Pr(>|t|)
lag 5.49568 0.73313 7.496 8.75e-07
mumax 0.50478 0.04104 12.299 6.90e-10
LOG10N0 4.43222 0.08610 51.476 < 2e-16
LOG10Nmax 7.19888 0.04305 167.216 < 2e-16

t-based confidence interval:
2.5% 97.5%
lag 3.9489102 7.0424564
mumax 0.4181883 0.5913768
LOG10N0 4.2505607 4.6138828
LOG10Nmax 7.1080516 7.2897126</td><td>Parameters:
Estimate Std. Error t value Pr(>|t|)
lag 6.77638 0.60001 11.29 2.53e-09
mumax 0.70342 0.06918 10.17 1.21e-08
LOG10N0 4.42748 0.08306 53.30 < 2e-16
LOG10Nmax 7.22436 0.04354 165.93 < 2e-16

t-based confidence interval:
2.5% 97.5%
lag 5.5104637 8.042298
mumax 0.5574642 0.849377
LOG10N0 4.2522273 4.602727
LOG10Nmax 7.1325033 7.316225</td></tr>
<tr><td>250-A</td><td>Parameters:
Estimate Std. Error t value Pr(>|t|)
lag 5.02311 1.32142 3.801 0.00143
mumax 0.41737 0.04216 9.901 1.79e-08
LOG10N0 4.75066 0.11165 42.551 < 2e-16
LOG10Nmax 7.23400 0.03843 188.231 < 2e-16

t-based confidence interval:
2.5% 97.5%
lag 2.2351605 7.8110672
mumax 0.3284284 0.5063104
LOG10N0 4.5151020 4.9862126
LOG10Nmax 7.1529141 7.3150809</td><td>Parameters:
Estimate Std. Error t value Pr(>|t|)
lag 4.01942 0.75888 5.297 5.92e-05
mumax 0.32768 0.01759 18.624 9.56e-13
LOG10N0 4.85089 0.07402 65.537 < 2e-16
LOG10Nmax 7.21801 0.03156 228.698 < 2e-16

t-based confidence interval:
2.5% 97.5%
lag 2.4183304 5.6205160
mumax 0.2905554 0.3647983
LOG10N0 4.6947282 5.0070556
LOG10Nmax 7.1514187 7.2845956</td><td>Parameters:
Estimate Std. Error t value Pr(>|t|)
lag 5.36841 1.11498 4.815 0.000162
mumax 0.43335 0.04859 8.919 8.05e-08
LOG10N0 4.84063 0.11253 43.017 < 2e-16
LOG10Nmax 7.26117 0.05388 134.768 < 2e-16

t-based confidence interval:
2.5% 97.5%
lag 3.0160183 7.7208104
mumax 0.3308403 0.5358573
LOG10N0 4.6032156 5.0780393
LOG10Nmax 7.1474982 7.3748481</td></tr>
<tr><td>250-B</td><td>Parameters:
Estimate Std. Error t value Pr(>|t|)
lag 6.84676 0.80604 8.494 1.60e-07
mumax 0.54597 0.03973 13.741 1.23e-10
LOG10N0 4.17156 0.08754 47.651 < 2e-16
LOG10Nmax 7.30172 0.03873 188.531 < 2e-16

t-based confidence interval:
2.5% 97.5%
lag 5.1461656 8.5473472
mumax 0.4621443 0.6298044
LOG10N0 3.9868618 4.3562666
LOG10Nmax 7.2200127 7.3834368</td><td>Parameters:
Estimate Std. Error t value Pr(>|t|)
lag 5.70725 0.58307 9.788 2.12e-08
mumax 0.45295 0.02329 19.451 4.72e-13
LOG10N0 4.21579 0.06346 66.430 < 2e-16
LOG10Nmax 7.28594 0.03314 219.840 < 2e-16

t-based confidence interval:
2.5% 97.5%
lag 4.4770777 6.9374263
mumax 0.4038168 0.5020795
LOG10N0 4.0819007 4.3496879
LOG10Nmax 7.2160212 7.3558685</td><td>Parameters:
Estimate Std. Error t value Pr(>|t|)
lag 7.39798 0.79952 9.253 4.77e-08
mumax 0.60156 0.05993 10.037 1.47e-08
LOG10N0 4.25260 0.09963 42.685 < 2e-16
LOG10Nmax 7.34809 0.06038 121.688 < 2e-16

t-based confidence interval:
2.5% 97.5%
lag 5.7111350 9.084817
mumax 0.4751119 0.728002
LOG10N0 4.0424034 4.462793
LOG10Nmax 7.2206893 7.475490</td></tr>
<tr><td>250-C</td><td>Parameters:
Estimate Std. Error t value Pr(>|t|)
lag 7.98046 1.47260 5.419 4.60e-05
mumax 0.45338 0.05833 7.772 5.39e-07
LOG10N0 4.59754 0.12645 36.359 < 2e-16
LOG10Nmax 7.28125 0.06318 115.248 < 2e-16

t-based confidence interval:
2.5% 97.5%
lag 4.8735405 11.0873883
mumax 0.3303127 0.5764509
LOG10N0 4.3307569 4.8643238
LOG10Nmax 7.1479497 7.4145409</td><td>Parameters:
Estimate Std. Error t value Pr(>|t|)
lag 4.70389 1.21316 3.877 0.00121
mumax 0.33258 0.02555 13.017 2.87e-10
LOG10N0 4.52266 0.12722 35.549 < 2e-16
LOG10Nmax 7.25510 0.05690 127.517 < 2e-16

t-based confidence interval:
2.5% 97.5%
lag 2.144336 7.2634368
mumax 0.278674 0.3864879
LOG10N0 4.254247 4.7910758
LOG10Nmax 7.135062 7.3751394</td><td>Parameters:
Estimate Std. Error t value Pr(>|t|)
lag 8.54368 1.32259 6.460 5.88e-06
mumax 0.47570 0.07461 6.376 6.90e-06
LOG10N0 4.69993 0.12678 37.071 < 2e-16
LOG10Nmax 7.32816 0.09054 80.940 < 2e-16

t-based confidence interval:
2.5% 97.5%
lag 5.7532597 11.3341000
mumax 0.3182869 0.6331197
LOG10N0 4.4324425 4.9674133
LOG10Nmax 7.1371399 7.5191790</td></tr>
</table>

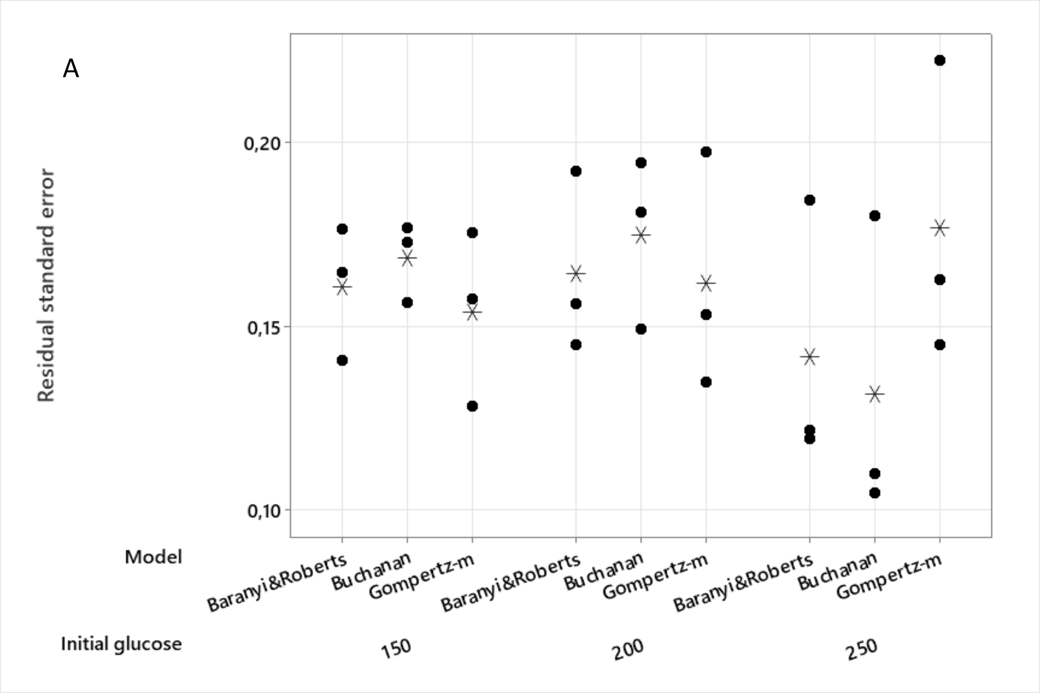

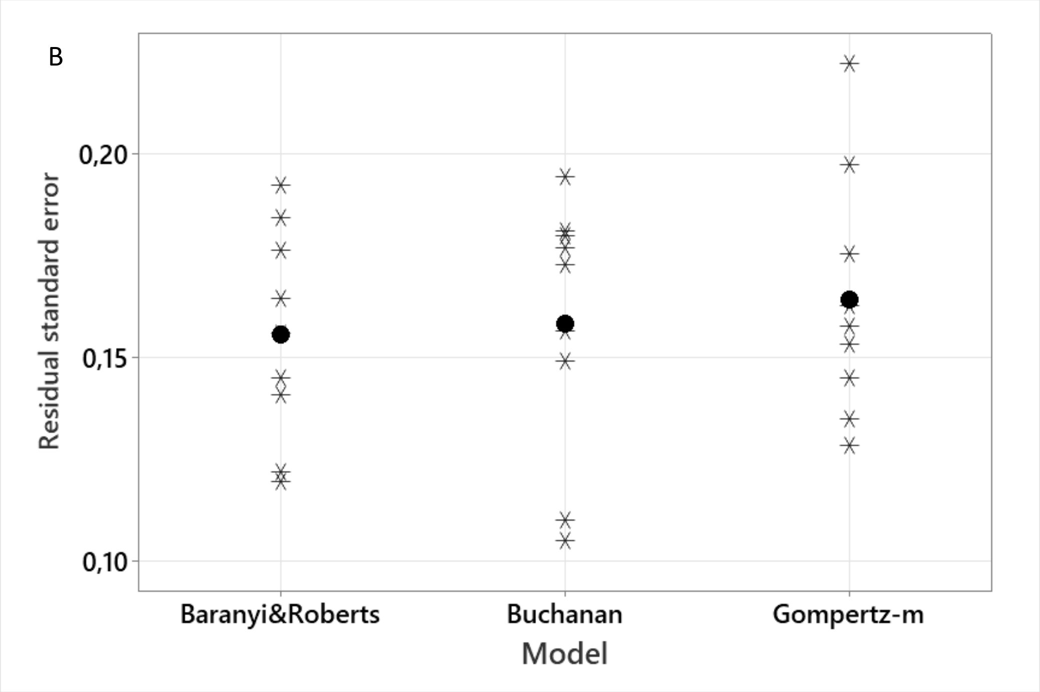

Figure S4. Residual standard error of the three growth models (Baranyi&Roberts, Buchanan, and Gompertz-m) considering (A) or disregarding (B) the initial glucose concentration of the cultures.

(a)

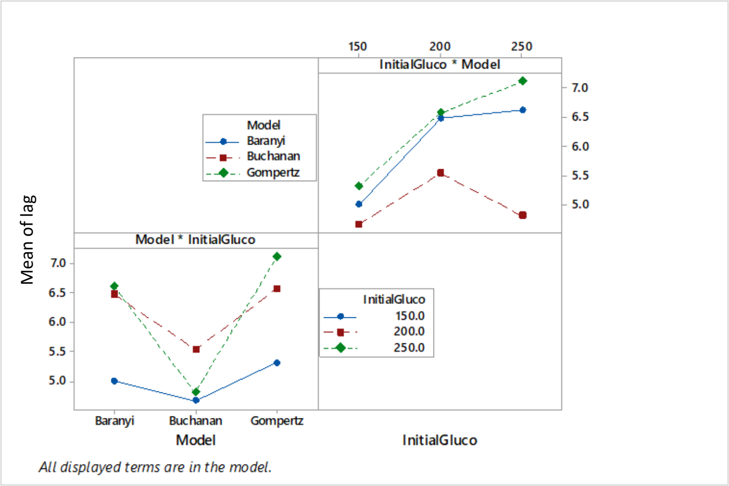

(b)

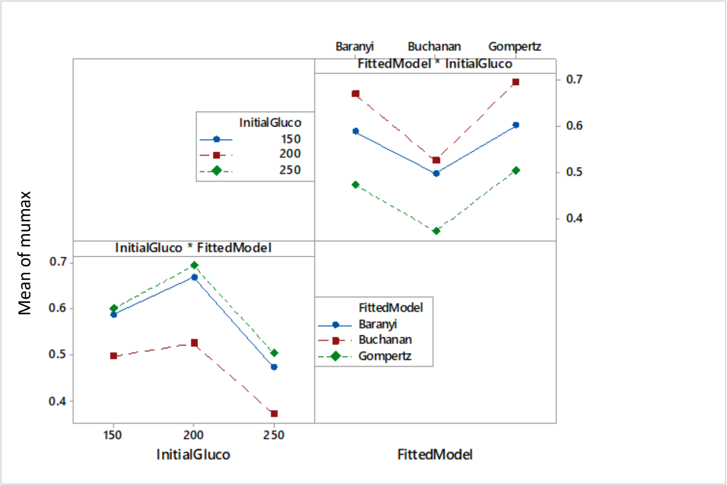

(c)

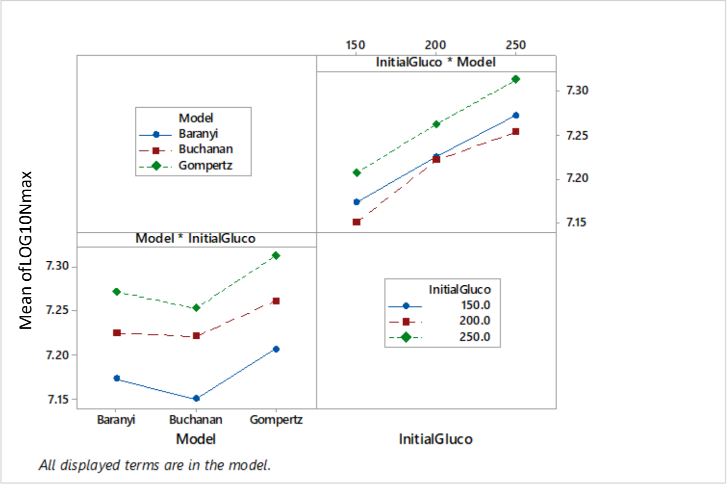

Figure S5. Interaction plots of the of the ANOVA analysis conducted over kinetic parameters estimated with the three models (Barany&Roberts, Buchanan, and Gompertz-m), (a) duration of the lag phase (lag), (b) maximum specific growth rate (mumax), and (c) maximum population density (LOG10max), on three initial glucose concentrations (150, 200 and 250 g/L).

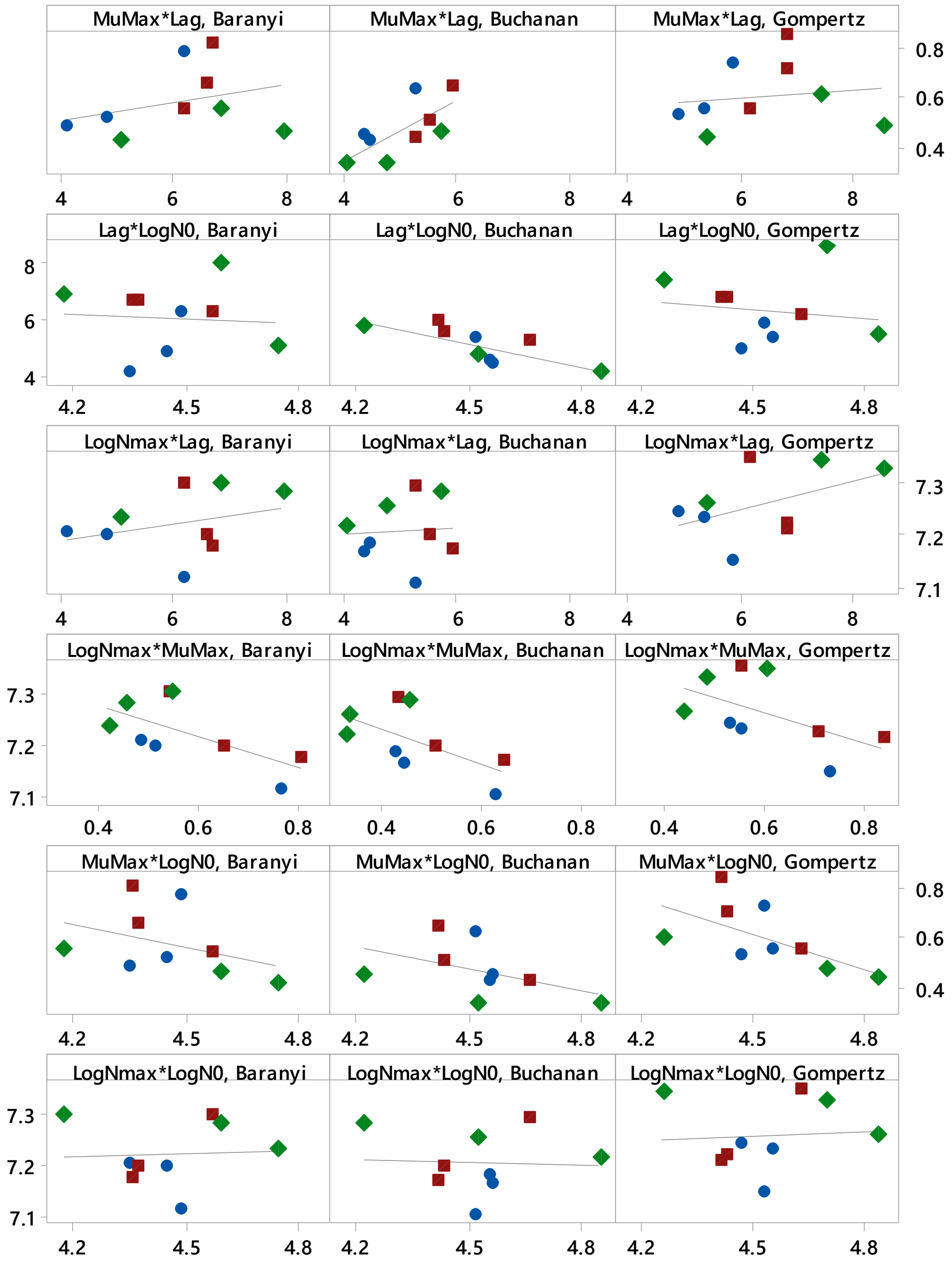

Figure S6. Scatter diagrams for the estimates obtained for the kinetic parameters, duration of the adaptation or lag phase (Lag), maximum specific growth rate (MuMax), and initial population and final population (LogN0 and LogNmax, respectively), according to the model used (Baranyi&Roberts, Buchanan, Gompertz-m). The three initial glucose concentrations 150 (blue circles), 200 (red squares), and 250 g/L (green diamonds) are shown.

Linear regression study between pairs of estimates obtained with alternative growth models

Table S2 provides the outputs of the linear regression study conducted between pairs of parameter estimates obtained from alternative models. The table provides the coefficients of the linear correlations with their significance, in addition to information about the fitted regression lines, the p-value of the statistical test applied to conclude whether or not it can be rejected that the term of the ordinate at the origin of the fitted line equals zero, and the confidence intervals for the two terms of the regression line, the independent term (ordinated to the origin) and the slope of the line. These outputs allow to fundamentally conclude whether the estimates for the terms of the linear relationship, independent term, and slope of the correlations can be assumed equal to 0 or to 1, respectively.

All the linear correlation coefficients obtained between the estimates made for each of the 4 kinetic parameters have been above 0.90 apart from the lag parameter. For the lag parameter, a very good linear correlation has been observed between estimates made with the Gompertz-m and Baranyi&Roberts models. Correlation between lag values estimated by Buchanan and Baranyi&Roberts has been low. Likewise, correlation between lag values estimated by Gompertz-m and Buchanan has been very low.

Considering the estimations by confidence intervals, for the lag phase duration, we could accept that estimates made with different linear models are not significantly different except in two cases, when the estimate made by the Buchanan model is obtained from the estimates made with the Baranyi&Roberts model or with the Gompertz-m model. In these two cases the non-concordance of values in the estimates made is quite relevant since the differences at the origin are around 3 hours and the slope of the line is significantly lower than 1.

In the case of the specific growth rate (mumax), it can be accepted that the estimates made using alternative models are not significantly different except for the case of Buchanan and Baranyi&Roberts' estimates.

For the initial population density (LOG10N0) parameter, no significant differences are detected considering the three adjustment models. Regarding the maximum population density (LOG10Nmax), no significant differences between models are detected either, with the exception of the case combining the values of the Gompertz-m and Baranyi&Roberts models, where it can be concluded that the two linear regression lines obtained have ordinates in the origin significantly different from zero (there is a translation of values) and where the slopes are significantly different from 1 (a conversion factor must be considered).

Table S2. The table includes: (i) Pearson correlations and p-Values (in brackets); (ii) linear regression lines to show the relationship between the estimates of the four kinetic parameters studied (duration of the lag phase (lag), maximum specific growth rate (mumax), initial population density (LOG10N0), and final population density (LOG10Nmax)) obtained with the three models used; (iii) significance (p-value) of the contrast for a value equal to or different from 0 of the ordinate at the origin of the regression line, and (iv) the confidence interval for the estimated intercept and the estimated slope of the regression line. Bu: Buchanan model, Gom: Gompertz-m model, B&R: Baranyi&Roberts model.

	Pearson Correlation Coefficient (p-value)		Regression line	P-Value for the Intercept test =0 *vs* ≠0	95% Confidence Interval for the intercept and for the slope
	lag-B&R	lag-Bu			
lag-Bu	0.607<br>(0.083)		lag-Bu = 3.00 + 0.333 lag-B&R	0.021	(0.61, 5.39)<br>(-0.056, 0.722)
lag-Gom	0.957<br>(0.000)	0.493<br>(0.178)	lag-Gom = 0,74 + 0,927 lag-B&R	0.296	(-0.80, 2.28)<br>(0.676, 1.178)
			lag-Gom = 1,97 + 0,871 lag-Bu	0.670	(-4.97, 8.91)<br>(-0.504, 2.247)
			lag-B&R = 0,48 + 1,108 lag-Bu	0.866	(-6.06, 7.02)<br>(-0.188, 2.405)
			lag-B&R = - 0,22 + 0,988 lag-Gom	0.771	(-1.94, 1.50)<br>(0.720, 1.255)
			lag-Bu = 3,24 + 0,279 lag-Gom	0.030	(0.42, 6.07)<br>(-0.161, 0.718)
	mumax-B&R	mumax-Bu			
mumax-Bu	0.978		mumax-Bu = 0,0158 + 0,779 mumax -B&R	0.684	(-0.0720, 0.1035)

	(0.000)				(0.6300, 0.9271)
mumax-Gom	0.976<br>(0.000)	0.963<br>(0.000)	mumax -Gom = 0,0697 + 0,920 mumax -B&R	0.170	(-0.0382, 0.1777)<br>(0.7372, 1.1027)
			mumax -Gom = 0,0707 + 1,139 mumax -Bu	0.260	(-0.0658, 0.2072)<br>(0.852, 1.426)
			mumax -B&R = 0,0057 + 1,228 mumax -Bu	0.907	(-0.1057, 0.1172)<br>(0.9941, 1.4628)
			mumax -B&R = - 0,0451 + 1,036 mumax -Gom	0.425	(-0.1711, 0.0808)<br>(0.8301, 1.2417)
			mumax -Bu = - 0,0233 + 0,813 mumax -Gom	0.673	(-0.1487, 0.1020)<br>(0.6083, 1.0179)
	LOG10N0-B&R	LOG10N0-Bu			
LOG10N0-Bu	0.906<br>(0.001)		LOG10N0-Bu = 0.367 + 0.933 LOG10N0-B&R	0.634	(-1.375, 2.109)<br>(0.542, 1.323)
LOG10N0-Gom	0.988<br>(0.000)	0.915<br>(0.001)	LOG10N0-Gom = 0.020 + 1.013 LOG10N0-B&R	0.944	(-0.613, 0.652)<br>(0.871, 1.155)
			LOG10N0-Gom = 0.415 + 0.910 LOG10N0-Bu	0.566	(-1.213, 2.042)<br>(0.551, 1.270)
			LOG10N0-B&R = 0.479 + 0.879 LOG10N0-Bu	0.518	(-1.187, 2.145)<br>(0.511, 1.247)
			LOG10N0-B&R = 0.088 + 0.964 LOG10N0-Gom	0.744	(-0.524, 0.700)<br>(0.829, 1.098)
			LOG10N0-Bu = 0.358 + 0.919 LOG10N0-Gom	0.623	(-1.289, 2.004)<br>(0.556, 1.282)
	LOG10Nmax-B&R	LOG10Nmax-Bu			
LOG10Nmax-Bu	0.986<br>(0.000)		LOG10Nmax-Bu = 0.167 + 0.975 LOG10Nmax-B&R	0.726	(-0.915, 1.249)<br>(0.825, 1.125)
LOG10Nmax-Gom	0.997<br>(0.000)	0.980<br>(0.000)	LOG10Nmax-Gom = - 0.6267 + 1.09 LOG10Nmax-B&R	0.041	(-1.217, -0.035)<br>(1.010, 1.174)
			LOG10Nmax-Gom = - 0.562 + 1.085 LOG10Nmax-Bu	0.384	(-1.994, 0.870)<br>(0.887, 1.284)
			LOG10Nmax-B&R = 0.041+ 0.996 LOG10Nmax-Bu	0.932	(-1.063, 1.145)<br>(0.843, 1.150)
			LOG10Nmax-B&R = 0.620 + 0.909 LOG10Nmax-Gom	0.021	(0.125, 1.115)<br>(0.841, 0.978)
			LOG10Nmax-Bu = 0.787 + 0.884 LOG10Nmax-Gom	0.157	(-0.388, 1.962)<br>(0.722, 1.046)
